# The mouse Social Frailty Index (mSFI): a novel behavioral assessment for impaired social functioning in aging mice

**DOI:** 10.1007/s11357-024-01263-4

**Published:** 2024-07-11

**Authors:** Charles W. Collinge, Maria Razzoli, Rachel Mansk, Seth McGonigle, Dudley W. Lamming, Christina A. Pacak, Ingrid van der Pluijm, Laura Niedernhofer, Alessandro Bartolomucci

**Affiliations:** 1 Department of Integrative Biology and Physiology, University of Minnesota, Minneapolis, MN USA; 2 Department of Medicine, University of Wisconsin, Madison, WI USA; 3 William S. Middleton Memorial Veterans Hospital, Madison, WI USA; 4 Greg Marzolf Jr. Muscular Dystrophy Center & Department of Neurology, University of Minnesota, Minneapolis, MN USA; 5 Department of Molecular Genetics, and Department of Vascular Surgery, Cardiovascular Institute, Erasmus University Medical Center, Rotterdam, The Netherlands; 6 Department of Biochemistry, Molecular Biology and Biophysics, University of Minnesota, Minneapolis, MN USA; 7 Institute on the Biology of Aging and Metabolism, University of Minnesota, Minneapolis, MN USA

**Keywords:** Clinical frailty index, Biological age, Social determinants of health, Geroscience, Progeria

## Abstract

Various approaches exist to quantify the aging process and estimate biological age on an individual level. Frailty indices based on an age-related accumulation of physical deficits have been developed for human use and translated into mouse models. However, declines observed in aging are not limited to physical functioning but also involve social capabilities. The concept of “social frailty” has been recently introduced into human literature, but no index of social frailty exists for laboratory mice yet. To fill this gap, we developed a mouse Social Frailty Index (mSFI) consisting of seven distinct assays designed to quantify social functioning which is relatively simple to execute and is minimally invasive. Application of the mSFI in group-housed male C57BL/6 mice demonstrated a progressively elevated levels of social frailty through the lifespan. Conversely, group-housed females C57BL/6 mice manifested social frailty only at a very old age. Female mice also showed significantly lower mSFI score from 10 months of age onward when compared to males. We also applied the mSFI in male C57BL/6 mice under chronic subordination stress and in chronic isolation, both of which induced larger increases in social frailty compared to age-matched group-housed males. Lastly, we show that the mSFI is enhanced in mouse models that show accelerated biological aging such as progeroid *Ercc1*^−/Δ^ and *Xpg*^−*/*−^ mice of both sexes compared to age matched littermate wild types. In summary, the mSFI represents a novel index to quantify trajectories of biological aging in mice and may help elucidate links between impaired social behavior and the aging process.

**Supplementary information:**

The online version contains supplementary material available at 10.1007/s11357-024-01263-4.

## Introduction

The simultaneous evolution of public health practices, medicine, technology, and sociopolitical systems has given rise to rapidly aging populations around the globe [1–3]. Ubiquitously characterized by declines in health and overall functioning, the aging process itself represents the single largest risk factor for chronic diseases and mortality [4, 5]. Advances in biomedical research to determine the causal factors underlying this phenomenon have fueled the advent of geroscience, galvanizing a pursuit to understand the natural history and biological roots of the aging process [6–8]. To foster this understanding, methods to reliably quantify the aging process at the individual level must first be established.

Despite being an inexorable aspect of human experience, the aging process is not homogenous in how it manifests. Individuals of the same chronological age may experience chronic diseases and age-related morbidities, whereas others remain relatively healthy [9–12]. Methods that seek to quantify the quality of one’s biological aging should capture heterogeneity and plasticity within the natural aging process while better characterizing the “pace” of aging on the individual level [13–16].

Besides algorithms based on DNA methylation or other plasma biomarkers, frailty has emerged as a prevalent outcome in the quantification of the biological aging process, becoming standard measure in research and geriatric clinical practice [10, 17]. An age-related state of increased vulnerability to adverse health outcomes, frailty is the cumulative result of the multilevel biological decline that occurs during aging [18, 19]. Indices based on an accumulation of health deficits over time can be constructed as an easy and reliable way to measure frailty as a summary of biological aging in human clinical populations [20–23]. Frailty indices show high predictive validity for age-related morbidity and mortality outcomes, therefore functioning as a robust indicator of biological aging [24–28]. Furthermore, physical frailty indices based on the accumulation of health deficits have been translated to application in mice, such as the 31-Item Clinical Frailty Index (CFI) [29]. The CFI shows high inter-rater reliability and promising translatability, and was recently used to generate algorithms accurately predictive of chronological age and life expectancy in mice [29–33].

Hitherto, the quantification of frailty in deficit accumulation-based indices has utilized parameters that are predominantly physical in nature. Yet, many of the factors that influence healthspan and mediate the pace of biological aging in both humans and animals are social in nature and often associated with chronic stress [34–38]. This notion makes it imperative to integrate a biological and physical account of the aging process with social science, and vice versa [37, 39–41]. Social behavior represents the interface between underlying psychological functions, neuroendocrine systems, and the social environment. Thus, one’s social capabilities may be a potential mediator between social determinants and their effects on health and lifespan [34, 37, 42]. Nevertheless, parameters describing social capabilities are not widely included or heavily weighted in deficit accumulation-based frailty indices [15, 43–45]. To account for this absence, models that strive to integrate the social manifestations of frailty into its conceptualization and measurement have been proposed [46–51]. “Social frailty”—an age-related state defined by a lack of social skills and resources necessary to achieve well-being, conferring greater risk of morbidity and mortality—has emerged as a separate but parallel concept to traditional physical frailty models, is gaining popularity [47], and has been shown to predict 4-year mortality in people aged 65 years and older [52].

Although the impacts of some social determinants on longevity have been recapitulated in mice, and evolutionarily conserved declines in social capabilities during the aging process have been observed, there is no method to quantify the social aspects of frailty in laboratory mice [37, 53–57]. Thus, we sought to develop a novel index of social frailty for mouse studies. Based on deficit accumulation rationale and optimized for use in longevity studies, the mouse Social Frailty Index (mSFI) non-invasively measures essential facets of social behavioral functioning in mice. The mSFI is composed of seven socio-behavioral assays (see Methods for details): Item no. 1, olfactory test, evaluates the ability of mice to discriminate between social and nonsocial odors; items no. 2 and no. 3, urine marking and countermarking in response to a mixed sex urine, evaluate spontaneous marking behavior known to release pheromones used for social communication and establishment/defense of a territory; items no. 4 and no. 5, juvenile social interaction in home cage or novel cage, evaluate social interactions toward a same-sex juvenile; item no. 6, social/novel object preference test, evaluates preference for a social stimulus vs. a novel object in a novel environment; and finally item no. 7, nest building, evaluates motivation and ability to build a nest—that, being important for heat conservation and shelter, is also foundational for huddling of breeding pairs or social groups, representing the privileged site for social communication with newborns, and it is typically the core of the territory by both male and female mice defended again intruders [58–61].

We first developed and validated the index with naturally aging male and female C57BL/6 mice from the National Institute on Aging (NIA) aged rodent colony, and compared the mSFI with the classical CFI in the same mice. Next, we tested the sensitivity of the mSFI to manipulations of the social environment known to elicit variable levels of stress in male mice: chronic social isolation, known to cause exaggerated behavioral responses to novelty and increased disease vulnerability [62, 63]; chronic subordination stress, a model exacerbating and anticipating the onset of age-related diseases, and shortening lifespan [42, 56, 57]. Finally, *Ercc1*^−/Δ^ and *Xpg*^−*/*−^ strains were used to validate the mSFI in progeroid models. Both genetic models present multisystem segmental progeria phenotypes achieved through an accumulation of DNA damage due to deficits in DNA damage repair pathways [64–67]. *Ercc1*^−/Δ^ mice possess one null ERCC1 allele and one truncated allele, and express ~ 5% of the normal level of ERCC1/XPF [68, 69]. *XPG*^−*/*−^ mice are deficient in XPG (ERCC5), the endonuclease responsible for the 3′ incision on the damaged DNA strand, which also has roles in nucleotide excision repair [64, 70, 71]. *Ercc1*^−/Δ^ mice have a maximum lifespan of 26–29 weeks while *XPG*^−*/*−^ mice have a maximum lifespan of 16–18 weeks [64, 65].

Cumulatively, our results provide robust evidence that social functioning can be quantified using our novel index, is impaired in naturally aging mice, and can be worsened by both psychosocial manipulations and progeria syndromes.

## Methods

### Animals

Group-housed male and female C57BL/6 mice ages 4–36 months were obtained from the NIA Aged Rodent Colony, housed in unisexual groups of three to five mice and acclimatized to our animal facility for 10–14 days. The 21-month-old experimental time point refers to group-housed mice of an age ranging from 20 to 22 months of age, 27 months represents mice aged 26–28 months, and 34 months represents mice aged 32–36 months. Two-month-old male and female *Xpg*^−*/*−^ mice along with age-matched wild-type (wt) were generated as previously described, housed in groups of three to five littermates [64]. Two-month-old male and female *Ercc1*^−/Δ^ mice along with age-matched wt mice were generated as previously described and housed in groups of three to five littermates [69]. *Ercc1*^−/Δ^ mice are in an F1 hybrid FVB/C57BL/6 J background obtained by crossing *Ercc1*^*−/*+^ with *Ercc1*^*Δ/*+^ mice of C57BL/6 J and FVB background. Four-month-old male C57BL/6 mice used as experimental mice in individual housing, chronic subordination stress (CSS), or lifelong chronic subordination stress (LCSS) paradigms were bred from existing stock (Jackson Laboratory, Bar Harbor, ME) and aged until the points specified. Retired male CD1 breeders were purchased from Charles River Laboratories (Wilmington, MA) to serve as resident dominants in experiments that used CSS and LCSS paradigms. The specific number of mice used in each experiment is indicated in the result section. Unless otherwise stated, all mice were housed in static cages with standard bedding and nesting material on a 12:12-h light/dark cycle at 21 $\pm$ 2°C and had access to standard diet (2018 Teklad; Inotiv, Madison, WI; Details for the NIA mice can be found here https://www.nia.nih.gov/research/dab/aged-rodent-colonies#barrier_information) and water ad libitum. All mice were cared for and maintained according to ethical guidelines set by the US National Institutes of Health. Experiments were approved by the Institutional Animal Care and Use Committee (IACUC) at the University of Minnesota-Twin Cities (Minneapolis, MN) or the William S. Middleton Memorial Veterans Hospital (Madison, WI).

### The mouse Social Frailty Index

To assess and quantify the social behavioral functioning of experimental mice, a mSFI was developed based on a set of seven behavioral assays. Assays were chosen for being based on the spontaneous manifestation of mouse social behavior, commonly used, minimally disruptive, relatively high-throughput, and easily executed. All procedures of the mSFI took place in the room in which experimental mice were housed and therefore no room habituation before testing was required. The sequence of the mSFI is flexible by design: individual assays were conducted in a nonspecific and varying order during two sessions per day over the span of 3 days and one session (seventh assay) on the fourth day. All morning sessions started between 8:30 and 9:00 am and afternoon sessions, besides those used for nest building tests (see item no. 7), started between 1:00 and 1:30 pm. Assays included in the mSFI took place in either the home environment (olfactory test, juvenile social interaction in home environment, nest building) or novel environments (urine marking, urine countermarking, juvenile social interaction in a novel environment, social approach/avoidance—see individual item sections below for specifics). For assays in the home environment, mice housed in groups were randomly assigned to undergo testing in their home environment one at a time, while littermates were temporarily housed in adjacent cages. To test mice in subordination protocols (e.g., CSS or LCSS), CD1 mice were removed from the shared home cage and housed in temporary cages while experimental mice underwent testing. A detailed standard operating procedure protocol can be found as a Supplementary Appendix to this paper.

#### Item no. 1: olfactory test

To evaluate the ability of mice to spontaneously discriminate between social odors and nonsocial odors, a modification of the olfactory habituation/dishabituation test was used [72, 73]. Water as an odorless reference, pure vanilla extract diluted in water to 1:10000 as an attractive nonsocial odor [73], and a pooled mixture of intact male and female urine purchased from a commercial vendor (Biochemed Pharmacologicals, Winchester, VA) as a social odor were embedded in the cotton tips of 15 cm cotton swab applicators. Odors were then each presented for 2 min in the same sequence—water, vanilla, urine—to experimental mice with a 1-min interval between presentations. At the start of each testing interval, freshly embedded cotton swab applicators were inserted 4 cm into the wire cage top near the water bottle insert. The time mice spent engaging with the odor was then recorded by observers with a stopwatch. Engaging behavior was operationally defined as follows: the mouse’s nose is within 2 cm of the cotton swab, oriented towards the swab, and making visible efforts to inhale the odor; the mouse is chewing, licking, and/or gnawing at the swab; the mouse is holding the swab with its forepaws. To quantify social odor discrimination, a *% urine preference* was calculated as follows: amount of time spent sniffing urine (s) / total amount of time spent sniffing all odors (s) × 100.

#### Item no. 2: urine marking in novel environment

To quantify urine marking ability, we used a modified version of the Void Spot Assay [74, 75]. 153 $\pm 4$ mm × 292 $\pm 4$ mm sheets of Whatman™ 3MM chromatography blotting paper (Cytiva, Marlborough, MA) were inserted lining the bottom of novel static cages (186 mm × 298 mm × 128 mm). Experimental mice were then placed in lined novel cages and allowed to freely roam and deposit urinary marks for 1 h and then returned to their home cages. Chromatography paper was removed from the testing cages and dried overnight to allow for fixation of urinary marks. The marked sheets of chromatography paper were then fluoresced using a UV transilluminator (Ultra-Violet Products, Inc., San Gabriel, CA) and photographed with a high-resolution camera. The *number of urinary marks* deposited was quantified in individual fluorescent images utilizing ImageJ (Version 1.53 k; Wayne Rasband & NIH, Bethesda, MD) with a minimum threshold for marks of 100 pixels and a maximum threshold of 40,000 pixels to distinguish marking behavior from spot urination [76, 77].

#### Item no. 3: urine countermarking in novel environment

To quantify urine countermarking behavior, 10 µL of urine was centrally aliquoted onto Whatman™ 3MM chromatography paper (153 $\pm 4$ mm × 292 $\pm 4$ mm) to serve as a stimulus for urine marking [78, 79]. To standardize urine marking behavior, a pooled mixture of intact male and female urine from a commercial vendor (Biochemed Pharmacologicals, Winchester, VA) was used. Experimental mice were allowed to individually roam the cage and countermark on the paper for 1 h. Post-test processing and analysis of the countermarked paper were conducted exactly as described in the urine marking in novel environment test. The *no. of countermarkings* was quantified by including all urinary markings present that fell within the aforementioned dimensional thresholds, excluding the stimulus mark.

#### Item no. 4: juvenile social interaction in home environment

The procedure used to measure social interaction in the home environment was a modification of the resident-intruder test [80, 81]. A novel non-sexually mature sex- and strain-matched unfamiliar juvenile (3–4 weeks old) intruder from colony stock was placed in the cage with each experimental mouse. Mice were allowed to freely interact for 5 min, and their behaviors were recorded by experienced observers using instantaneous sampling every 5 s. The following three categories of social behavior exhibited by experimental mice were scored: sniffing behavior (pursuit, olfactory investigation, etc.), aggressive behavior (attack bites, sideways offensive posture, etc.), and allo-grooming behavior [81]. *% social interaction* was calculated as (total no. of social intervals) / (total no. of intervals observed) × 100.

#### Item no. 5: juvenile social interaction in novel environment

To assess levels of social behavior in a novel environment, we modified the procedure described in item no. 4. Experimental mice and same-sex unfamiliar juveniles were simultaneously placed in novel static cages (186 mm × 298 mm × 128 mm) with standard bedding, food, water, and allowed to freely roam for 5 min. Sniffing, aggression, and allo-grooming were measured with the instantaneous observational sampling method described in item no. 4. The *% social interaction* was calculated the same as in the home environment: (total no. of social intervals) / (total no. of intervals observed) × 100.

#### Item no. 6: social/novel object preference test

To assess the preference for a social stimulus vs. a novel object, a modified version of the three-chamber social approach task protocol was used [82, 83]. The testing apparatus was assembled in large static cages (257 mm × 483 mm × 152 mm) with standard bedding. A novel sex- and strain-matched unfamiliar juvenile and an inanimate object were placed under respective wire mesh cups and weighed down by a 500 mL water-filled bottle at opposite sides in each testing cage. Experimental mice were then placed in the cage for 5 min. At the end of each session, experimental mice were returned to their original home-cage, while objects and wire mesh cups were cleaned with 70% ethanol and allowed to dry before being reused. The instantaneous observational sampling method was used to track intervals spent interacting with the object and intervals spent interacting with the target juvenile mouse each defined as follows: actively sniffing or digging around the wire mesh cup; oriented towards it (e.g., stretch attend posture, rearing, or climbing on) the wire mesh cup containing either the juvenile or the object. The *% social approach* was calculated as (no. of intervals spent interacting with mouse – no. of intervals spent interacting with object) / total no. of intervals spent interacting × 100.

#### Item no. 7: nest building

Nests are important in heat conservation and shelter, but also serve an integral function in courtship and reproduction in mice [84, 85]. To quantify nest building ability, nest quality was assessed similarly to that previously described [86, 87]. Testing started approximately 1 h before the dark phase and concluded before the subsequent A.M. behavioral testing session. All Enviro-dri® nesting material was removed from the home cage of individually housed mice. If group-housed, mice were transferred into individual novel cages that included bedding from the original home cage. In the case of LCSS-housed mice, Enviro-dri® nesting material was removed from both sides of shared cages and dominant CD1 mice were temporarily individually housed overnight. These housing alterations were done to provide a controlled environment in which the nesting ability of individual mice could be measured and not confounded by cage-mates or CD1 dominant mice. One standard nestlet made of pressed cotton batting (Ancare Corp, Bellmore, NY) was placed centrally in experimental cages. Mice were allowed to build their nests overnight and nests were assessed the next morning. Nests were rated on the 5-point scale detailed in the Supplementary Appendix.

#### mSFI calculation

Each assay produces a quantitative score that represents behavioral performance in its respective dimension of social behavior (e.g., a % urine preference is calculated from the olfactory test). Similar to the standard procedure in deficit accumulation-based frailty indices and the 31-Item Clinical Frailty Index [23, 29], an mSFI from 0 to 1 was calculated for each mouse based on the extent of deviation from mean quantitative scores obtained in an initial reference sample of sex- and strain-matched young adult mice (3–4 months of age) that underwent mSFI testing. An mSFI of 0 represents an animal with no deficits in social functioning and an mSFI of 1 represents an animal with maximal deficits in social functioning. Between these minimal and maximal values exists a gradient of deficit levels with greater values representing greater social frailty. To calculate the mSFI, quantitative scores in each behavioral assay were assigned “index scores” as follows: scores less than $\pm$ 1 standard deviation (SD) of the reference population mean were assigned a 0, $\le$ 2 SD = 0.25, $\le$ 3 SD = 0.5, $\le$ 4 SD = 0.75, and $>$ 4 SD = 1. It should be noted that this methodology was not used in the case of the Nest Building assessment because it is scored on a 5-point scale. Instead, mSFI scores were assigned so that nest scores of 5 received an index score of 0, 4 = 0.25, 3 = 0.5, 2 = 0.75, and 1 = 5. Index scores for each mouse over all seven items were summed and divided by 7, the total number of items, to obtain a final mSFI value. mSFI values in the present study ranged from a minimum of 0.00 to a maximum of 0.57.

### 31-Item Clinical Frailty Index

To quantify physical frailty in experimental mice, the 31-Item Clinical Frailty Index was used [29]. In each experimental mouse, trained experimenters evaluated for signs of discomfort along with the integument, musculoskeletal, vestibulocochlear/auditory, ocular, nasal, digestive, urogenital, and respiratory systems via direct observation. Grip strength was evaluated using a grip strength meter (Columbus Instruments, Columbus, OH) and internal body temperature was taken anally with a temperature probe (Cole-Parmer, Vernon Hills, IL). Deficits in each observational item were scored based on criteria provided by Whitehead et al. (2014) using a simple scale: 0 = no sign of deficit, 0.5 = mild deficit present, and 1 = severe deficit present. Deficits in grip strength, internal body temperature, and body weight were scored based on deviation from a reference population of sex- and strain-matched young adult mice and assigned values with the same cutoffs used in the mSFI above. Deficit scores for all items were summed and divided by the total number of items (31) to calculate a final clinical frailty index value for each mouse. In all comparisons, clinical frailty assessments occurred within 24 h of the end of mSFI testing.

### Chronic subordination stress

Two cohorts of C57BL/6 experimental mice were subjected to two different chronic subordination stress (CSS) procedures starting at 4 months of age: 4-week CSS, and lifelong chronic subordination stress (LCSS) [56] lasting up to 21 months of age.

The 4-week CSS protocol was conducted as previously established [88–91]. Four-month-old experimental mice were individually housed in static cages and male CD1 mice were individually housed in large static cages (257 mm × 483 mm × 152 mm) during a baseline phase lasting 7 days to allow CD1 mice to establish territorial ownership. Immediately following the baseline phase, a stress phase ensued over 4 weeks consisting of daily social defeat sessions. Experimental mice were randomly assigned to CSS exposure or to serve as individually housed controls. CSS mice were placed as intruders in CD1 resident cages where they were allowed to freely interact for a maximum of 10 min while aggressive behavior exhibited by the CD1 and subordination postures manifested by experimental mouse were quantified. At the end of the interaction, the dyads were separated by a plexiglass wire mesh partition that allowed continuous sensory contact while restricting physical interaction during 24-h cohousing. Social defeat sessions occurred daily between 9:00 and 10:00 a.m. Baseline mSFI testing was conducted 4 weeks prior to the baseline phase of the 4-week CSS procedure as well as immediately after (day 29) the stress phase.

The LCSS model used in this study was conducted as previously described [56] and incorporates the CSS procedure above. After the CSS phase, however, mice entered an aging phase in which CD1 aggressor and experimental subordinate dyads were cohoused in continuous sensory contact with the partition in place in absence of physical social defeat for the remainder of their natural lifespan. Experimental mice in the LCSS model underwent mSFI testing at 10, 16, and 21 months of age.

### Statistical analysis

Statistical analyses were performed using R Studio (Version 4.3.0; Posit Software, PBC, Boston, MA) and GraphPad Prism (Version 10.2.0; GraphPad Software, LLC, San Diego, CA). Comparisons across age and housing groups were conducted via one-way ANOVA followed by Tukey’s HSD post hoc tests for individual binary comparisons. Comparisons containing more than one factor of analysis were conducted via two-way ANOVA followed by Šidák’s multiple comparison test to adjust the family-wise error rate in binary comparisons. Repeated measures one-way ANOVA was used to analyze the effect of age on mSFI values in individually housed and LCSS cohorts, and multiple imputation was utilized to account for missing values in the LCSS cohort (*N* = 3 values imputed at 21 months). Binary comparisons of individually housed and LCSS cohorts to 4-month-old mice by age group were tested using Šidák’s multiple comparison test. To calculate the mean contribution of each item to the overall mSFI value per mouse, individual index scores in each item were divided by the sum of a mouse’s index scores in all items, before multiplying that value by the mouse’s mSFI value. This was repeated in each item and contributions per item were averaged across mice in each age group. Relationships between mSFI and CFI values were assessed with Pearson’s *r*, linear, and non-linear least squares regression. Individual values were screened as outliers using the ROUT method before Akaike’s information criterion (AICc) was employed to determine best-fit models between mSFI and CFI values [92]. Instantaneous rates of change for best-fitting quadratic models were calculated by hand.

## Results

**Fig. 1:**
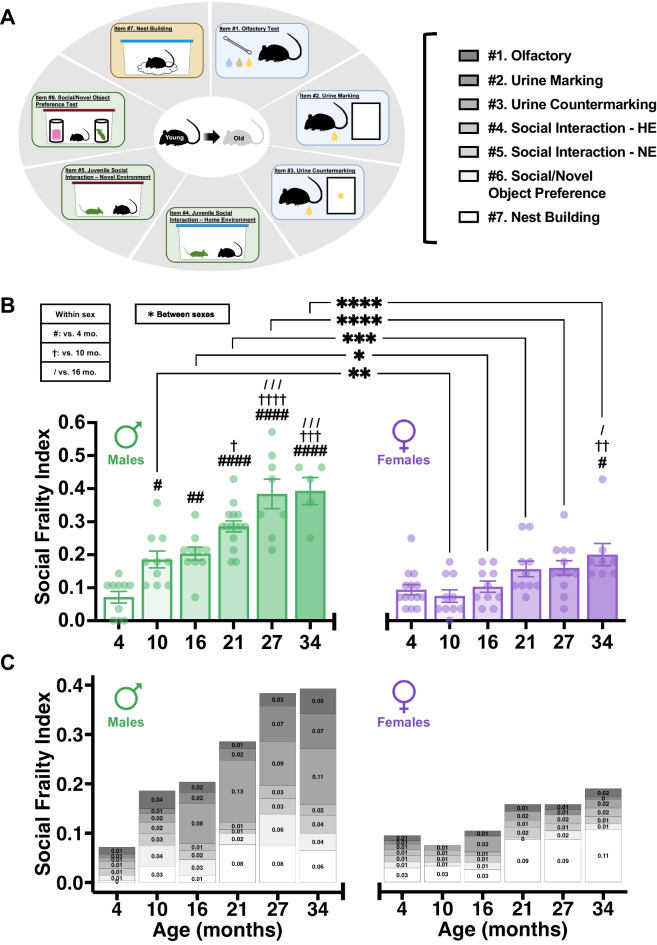
**A** Visual representation of assays that comprise the mSFI. Assays of social communication are shaded blue, social interaction assays are green, and the social motivation assay is shaded tan. **B** Mean $\pm$ SEM comparison of mSFI values with age in group-housed (GH) C57BL/6 male (green) and female (purple) mice. Significant effect of age on male mSFI value (one-way ANOVA; *F*(5, 52) = 20.47, *p* < .001)). Significant effect of age on female mSFI value (one-way ANOVA; *F*(5, 58) = 4.737, *p* = .001). # indicates significant difference vs. 4 months, † vs. 10 months, / vs. 16 months from Tukey’s HSD. Age-matched comparison of mSFI values by sex yielded significant main effects of sex (two-way ANOVA; *F*(1, 110) = 76.66, *p* < .001) and age (two-way ANOVA; *F*(5, 110) = 23.85, *p* < .001) on mSFI value in addition to sex × age interaction (two-way ANOVA; *F*(5, 110) = 6.84, *p* < .001). *Significant difference between sexes from Šidák’s multiple comparisons test. One symbol: *p* < .05; two symbols: *p* < .01; three symbols: *p* < .001; four symbols: *p* < .0001 post hoc. **C** Mean mSFI values with age presented as a composite of the mean value contributed by each individual item (note: mSFI range on *y*-axis constricted to zoom in on mean individual assay contribution values)

The objective of this study was to characterize a novel index of social behavioral impairments expected to accumulate during the aging process in mice, the mSFI (Fig. 1A). The mSFI is comprised of seven items, including assays of social communication, social interaction and motivation, and functional ability relevant for social bonding in addition to courtship and temperature regulation. To accomplish our objective, we first used a cross-sectional design in which the mSFI and the CFI were tested in group-housed male and female C57BL/6 mice of various ages obtained from the NIA aged rodent colony. To increase the generalizability of our measure to external aging studies, all mice were naturally aged in unisexual groups. Both indices were initially applied to a population of 4-month-old group-housed male (*N* = 10) and female (*N* = 14) mice to obtain the mSFI and CFI reference values; all subsequent applications of the mSFI and CFI on group-housed C57BL/6 mice in this study used these samples as a reference as described in the methods. The mean quantitative values and standard deviations obtained from these reference samples in each item of the mSFI are displayed in Supplementary Tables 1–2.

### Group-housed C57BL/6 male and female mice show age-related increases in social frailty: trajectories and sex differences

To determine if the mSFI quantifies age-related impairment in social behavioral functioning that accumulates throughout the lifespan as the biological aging process progresses, we tested it in male mice at 10 (*N* = 10), 16 (*N* = 10), 21 (*N* = 15), 27 (*N* = 8), and 34 (*N* = 5) months of age and in female mice at 10 (*N* = 10), 16 (*N* = 10), 21 (*N* = 10), 27 (*N* = 12), and 34 (*N* = 8) months of age. In C57BL/6 males, a clear gradient of increasing social frailty with age is apparent and supported by a significant effect of age on mSFI value (Fig. 1B, Supplementary Table 1). The earliest age at which a significant increase in social frailty was observed compared to 4-month-old mice occurred at 10 months of age, with mSFI values increasing significantly up to 27 months of age where they plateaued through values observed at 34 months of age. A significant effect of age on mSFI scores was found also in C57BL/6 female mice (Fig. 1B; Supplementary Table 2). However, the increase in female social frailty became significant only in the oldest age group assessed compared to the 4-month-old reference group.

We next sought to directly compare the age-related increases in social frailty across both sexes. The presence of sex differences in the progression of social frailty with age was supported by an age × sex interaction in addition to main effects of age and sex on mSFI values (Fig. 1B). No significant differences in mSFI values emerged between the male and female at 4 months of age (Fig. 1B). However, male mice exhibited significantly elevated mSFI values compared to age-matched female mice at 10, 16, 21, 27, and 34 months of age (Fig. 1B).

Because the mSFI contains seven items that quantify distinct aspects of social behavior, we analyzed the impact of age on how each item contributes to the composition of overall mSFi values (Fig. 1C; Supplementary Tables 1&2). In reference males at 4 months of age, the composition of mSFI values is relatively homogeneous, only varying by 0.01 between the lowest contributing item (Nest Building; 0.004) and the highest (Olfactory, Social Interaction–Home Environment & Novel Environment; 0.014) (Fig. 1C). Reference females at 4 months of age showed homogeneity in assays comprising the social communication and social interaction facets of the mSFI (Fig. 1C). The Nest Building assay had a relatively high contribution value in females at 4 months compared to other items. This high contribution from nest building remained through all ages of females measured. Visual inspection reveals that in comparison to females, males generally showed more impairment in assays that measure social communication and social interaction, although not across all ages measured.

Overall, these results show that social frailty worsens in progressively older group-housed C57BL/6 male mice; this occurs with a significantly greater severity than in group-housed female mice in which social frailty becomes significant only at the most advanced life stage tested.

### Contrasting age-related trajectories in mSFI and CFI in C57BL/6 mice

**Fig. 2:**
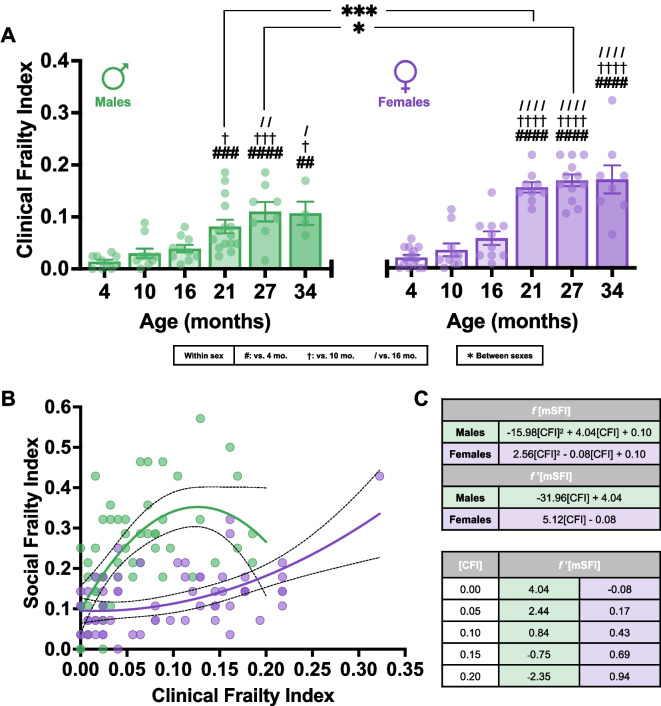
**A** Mean $\pm$ SEM comparison of CFI values with age in GH C57BL/6 male (green) and female (purple) mice. Significant effect of age on male CFI values (one-way ANOVA; *F*(5, 51) = 9.59, *p* < .001). Note: one 34-month-old male mouse died before CFI testing leaving a final *N* = 4 for this group. Significant effect of age on female CFI values (one-way ANOVA; *F*(5, 57) = 31.29, *p* < .001). Note: one 21-month-old female mouse died before CFI testing leaving a final *N* = 9 for this group. # portrays significant difference vs. 4 months, † vs. 10 months, / vs. 16 months from Tukey’s HSD. Age-matched comparison of CFI values by sex yielded significant main effects of sex (two-way ANOVA; *F*(1, 108) = 23.78, *p* < .001) and age (two-way ANOVA; *F*(5, 108) = 36.50, *p* < .001) on CFI value. Sex × age interaction (two-way ANOVA; *F*(5, 108) = 2.98, *p* = .015) was also found to be significant. *Significant difference between sexes from Šidák’s multiple comparisons test. One symbol: *p* < .05; two symbols: *p* < .01; three symbols: *p* < .001; four symbols: *p* < .0001 post hoc. **B** Individual mSFI values from all GH male and female mice plotted as a function of CFI values. CFI and mSFI values are strongly positively correlated in both males (*r*(55) = .52, *p* < .001) and females (*r*(61) = .51, *p* < .001). Data fit with curve describing the relationship between CFI and mSFI values from quadratic least-squares regression in males (green; [mSFI] =  − 15.98[CFI]^2^ + 4.04[CFI] + 0.10, *R*^2^ = .38, adjusted *R*^2^ = .36) and females (purple; [mSFI] = 2.56[CFI]^2^ – 0.08[CFI] + 0.10, *R*^2^ = .30, adjusted *R*^2^ = .28). Dashed lines represent 95% confidence intervals. **C** Instantaneous rates of change in mSFI values (*f*’[mSFI]) at CFI values relevant for both sexes based on quadratic regression

To compare the degree of social frailty quantified by the mSFI with a validated deficit accumulation-based method used to quantify age-related impairment as a measure of biological aging in mice, we administered the classical 31-tem Clinical Frailty Index (CFI) [29] to the same group-housed C57BL/6 male and female mice that underwent mSFI testing (Fig. 2A).

A significant increase in CFI values was observed with age in male mice (Fig. 2A). Notably, the earliest time point at which CFI values significantly increased when compared to reference 4-month-old mice was later than that observed in mSFI scores, occurring at 21 months of age, with values plateauing past 27 months of age. Unlike the delayed increase of mSFI values in females, significantly increased CFI values were observed as early as 21 months of age, which then plateaued after 27 months of age (Fig. 2A).

Consistent with previous literature [93], C57BL/6 females exhibited higher CFI values at 21 and 27 months of age compared to age-matched males (Fig. 2A). No significant differences in CFI values were apparent between the reference groups of 4 month-old males and females measured.

Both sensitive to chronological age, mSFI and CFI values were significantly and strongly positively correlated (Fig. 2B). Akaike information criterion (AICc) selection determined that the best fit model described a quadratic relationship between mSFI and CFI values both in males (Fig. 2B; ΔAICc vs. linear = 6.89, 96.9% cumulative model weight) and in females (Fig. 2B; ΔAICc vs. linear = 1.78, 70.89% cumulative weight). Nevertheless, the trajectories of these functions are very distinct (Fig. 2B). Both best-fit curves have identical intercept values (males 0.10, females 0.10) suggesting that at 4 months of age there is no intrinsic difference between social and physical frailty. However, male mice show steeply increasing instantaneous rates of change in mSFI values (*f’*[mSFI]) with initial increases in CFI values ([CFI] = 0.05; *f’*[mSFI] = 2.442), equating to high levels of social frailty at low levels of physical frailty (Fig. 2C). *f’*[mSFI] then declines with further increases in CFI up to [CFI] = 0.13, where *f’*[mSFI] reaches 0 at the inflection point of the best-fit quadratic. At CFI values thereafter, *f’*[mSFI] has a negative value through the maximum observed CFI value in males (0.19) to [CFI] = 0.20. The quadratic describing the relationship between mSFI and CFI values observed in females has opposite concavity compared to that of males and describes a much slower rate of social frailty increase with increasing CFI scores. At [CFI] = 0, females exhibit a slightly negative *f’*[mSFI], which reaches 0 immediately at the inflection point of the quadratic, [CFI] = 0.02. With subsequent increases in CFI value, mSFI values remain low before slowly and progressively increasing as *f’*[mSFI] consistently increases. Due to the less pronounced increases in mSFI value for each unit of CFI score increases, female mSFI values remain below those observed in males at similar CFI scores through the maximum observed CFI values in males.

Overall, although both the mSFI and CFI are sensitive to chronological age, our results also demonstrate that the two indices quantify biological aging differently between the two sexes. Group-housed C57BL/6 male mice appear to become socially frail at a younger age compared to females and increase physical frailty to a lesser degree than what is observed in females of comparable age after 21 months of age.

### The mSFI is insensitive to short-term social subordination stress exposure in male C57BL/6 mice

The mSFI is intended as a measure to quantify impairment in social behavior that occurs during the aging process rather than short-term social avoidance observed in response to experimental social manipulations. As chronic subordination stress (CSS; see Methods for details) in male mice has been documented to elicit social avoidance behavior in as little as 7 days up to 4 weeks, we aimed to examine the effects of CSS on the mSFI [94–97].

**Fig. 3:**
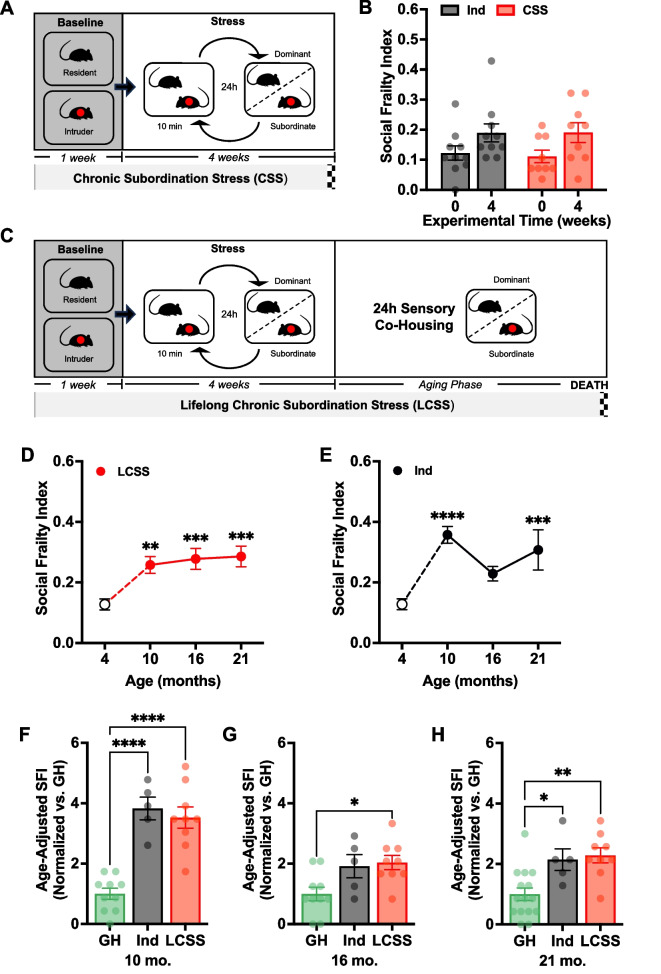
**A** Visual timeline of 4-week chronic subordination stress (CSS) procedure. Red dot represents experimental mouse experiencing chronic subordination stress. **B** Mean $\pm$ SEM comparison of mSFI values prior to baseline (Time = 0) and immediately after 4 weeks of control individual housing (Ind; *N* = 10) or CSS exposure (*N* = 9) in C57BL/6 male mice. No effect of treatment (two-way ANOVA; *F*(1, 17) = 0.04, *p* = .851), but a significant effect of time of measurement (two-way ANOVA baseline vs. post-social manipulation; *F*(1, 17) = 6.85, *p* = .018). No treatment × time interaction (two-way ANOVA; *F*(1, 17) = 0.05, *p* = .823). Despite main effect of time, no significant differences in mSFI values within treatment groups or between treatment groups at either time of measurement at the α = .05 level after Šidák correction. **C** Visual timeline of Lifelong Chronic Subordination Stress (LCSS) procedure consisting of the 4-week CSS procedure and a lifelong aging phase in established social hierarchy. **D** Mean $\pm$ SEM mSFI values with age in Ind C57BL/6 male mice (black) compared to 4-month Ind mice (white) that served as a reference sample to calculate the mSFI. Dashed lines represent hypothetical trajectory, solid lines represent repeated measures. No significant effect of age on mSFI values in aging Ind mice (one-way repeated measures ANOVA; *F*(1.40, 5.61) = 2.48, *p* = .171). **E** Mean $\pm$ SEM mSFI values with age in LCSS C57BL/6 male mice (red) compared to 4-month Ind mice (white). Dashed lines represent hypothetical trajectory, solid lines represent repeated measures. No significant effect of age on mSFI values in aging LCSS mice (one-way repeated measures ANOVA; *F*(1.85, 14.80) = 0.21, *p* = .798). **F**–**H** Mean $\pm$ SEM age-adjusted mSFI values of 10-month, 16-month, and 21-month Ind and LCSS-exposed C56BL/6 mice normalized to GH. **F** Significant effect of treatment on age-adjusted mSFI value (one-way ANOVA; *F*(2, 21) = 29.09, *p* < .001). **G** Significant effect of treatment on age-adjusted mSFI value (one-way ANOVA; *F*(2, 21) = 5.59, *p* = .011). **H** Significant effect of treatment on age-adjusted mSFI value (one-way ANOVA; *F*(2, 21) = 18.80, *p* < .001). *Significant difference from 4-month Ind males in Šidák’s multiple comparison test (Ind and CSS) or between treatment groups in Tukey HSD (GH vs. Ind vs. LCSS). One symbol: *p* < .05; two symbols: *p* < .01; three symbols: *p* < .001; four symbols: *p* < .0001

To do so, we applied the mSFI in individually housed 4-month-old C57BL/6 male mice (*N* = 20) prior to the start of a 4-week CSS protocol to obtain a baseline reference measurement from which to calculate the mSFI, and immediately following the protocol in mice that were randomly assigned to CSS-exposure (*N* = 9) or to individual housing (Ind) (*N* = 10) (Fig. 3A). It should be noted that one mouse in the CSS-exposed group died from causes unrelated to the physical aggression during the CSS protocol, leaving a final *N* = 9 for the CSS-exposed group. mSFI values at baseline did not significantly differ from those previously observed at 4 months of age in group-housed C57BL/6 males (*Individually housed* = 0.13 $\pm$ 0.02, *Group-housed* = 0.15 $\pm$ 0.02; unpaired *t*-test: *t*(28) = 0.55, *p* = 0.584). There were no differences in mSFI scores at baseline between future Ind and CSS-exposed (Fig. 3B: Ind; *baseline* = 0.12 $\pm$ 0.02, *treatment* = 0.19 $\pm$ 0.03 and CSS; *baseline* = 0.11 $\pm$ 0.02, *treatment* = 0.19 $\pm$ 0.03). Two-way repeated measures ANOVA revealed a significant main effect of time, but not of treatment or their interaction, implying that the slight increase in mSFI scores was due to a generalized increase in both control and CSS mice and not to within- or between-group differences (Fig. 3B). These results confirm that the mSFI is not attuned to phenotypes induced by short-term social manipulations, but rather social behavioral impairments that occur over the lifespan during the aging process which are relevant for geroscience.

### Lifelong chronic subordination stress and individual housing increase social frailty in male C57BL/6 mice

Extrapolating from our findings that the mSFI is not affected by 4-week CSS exposure and that the mSFI increases with chronological age in group-housed mice, we hypothesized that exposure to a model of lifelong chronic subordination stress (LCSS; Fig. 3C) or lifelong individual housing would result in progressive accelerated increases in mSFI scores with age compared to those observed in group-housed males.

To test this hypothesis, the mSFI was first applied on subordinate C57BL/6 male mice (*N* = 9) in an LCSS protocol at 10, 16, and 21 months of age. LCSS-exposed mice showed no significant changes in mSFI value from 10 to 21 months of age, but significantly elevated mSFI values compared to reference mice at 10, 16, and 21 months of age from the natural aging experiment presented in Fig. 1 (Fig. 3D).

We then applied the mSFI in lifelong individually housed C57BL/6 male mice (*N* = 5) at the same ages as LCSS-exposed mice. Similar to the LCSS, individually housed males also showed significant increases in mSFI values measured at 10 and 21, however not at 16 months of age when compared to those observed in reference mice from the experiment presented in Fig. 1 (Fig. 3E; Supplementary Fig. 1B). No significant differences in mSFI value from 10 to 21 months of age were found in lifelong individually housed males either.

As the males examined in LCSS, lifelong individual housing, and group housing conditions were independent experiments, they were not directly comparable statistically. Thus, we conducted a semi-quantitative comparison in which an age-adjusted mSFI was calculated for each age using the behavior of age-matched group-housed males in each of the seven individual assays composing the mSFI as the reference sample. Age-adjusted SFI scores were then normalized to the scores of age-matched group-housed mice from the experiment presented in Fig. 1, to account for differing absolute change from the group-housed baseline at each age. Albeit with limitations discussed above, this comparison reveals that LCSS male mice showed significantly elevated age-adjusted mSFI compared to group-housed males at all ages of our comparison while lifelong individually housed males showed significantly increased age-adjusted mSFI values compared to group-housed males at both 10 and 21 months of age (Fig. 3F–G). The age-adjusted mSFI values of individually housed male mice did not differ significantly from those of LCSS males at 10, 16, or 21 months of age.

Overall, both LCSS and lifelong individual housing increase mSFI values to levels that plateau after 10 months of age, but to an elevated extent when compared to group-housed males using a semi-quantitative comparison.

### Social and physical frailty are exacerbated in genetically induced progeria mouse models

**Fig. 4:**
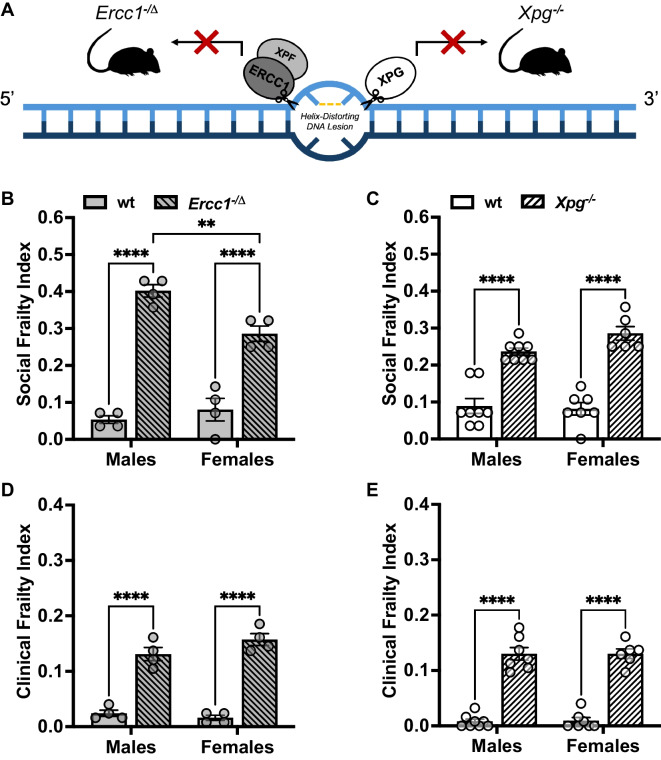
**A** In nucleotide excision repair (NER), XPG and ERCC1/XPF are recruited by the XPA-containing TFIIH complex to the 3′ and 5′ ends of the helix-distorting lesion, respectively. As part of strand-specific excision in NER, ERCC1/XPF incises the 5′ end of the helix-distorting lesion and XPG incises the 3′ end. *Ercc1*^−/Δ^ and *Xpg*^−*/*−^ mice commonly accumulate DNA-damage through ineffective NER pathways, leading to progeria. **B** Mean $\pm$ SEM mSFI values by sex and genotype in *Ercc1*^−/Δ^ mice vs. wt. Significant effect of genotype on mSFI value (two-way ANOVA; *F*(1, 12) = 174.70, *p* < .001), but no significant effect of sex (two-way ANOVA; *F*(1, 12) = 4.55, *p* = .054). Significant genotype × sex interaction (two-way ANOVA; *F*(1, 12) = 11.64, *p* = .005). **C** Mean $\pm$ SEM mSFI values by age and genotype in *Xpg*^−*/*−^ mice vs. wt. Significant effect of genotype on mSFI value (two-way ANOVA; *F*(1, 25) = 110.10, *p* < .001), but no effect of sex (two-way ANOVA; *F*(1, 25) = 1.53, *p* = .227) or genotype × sex interaction (two-way ANOVA; *F*(1, 25) = 2.87, *p* = .103). **D** Mean $\pm$ SEM CFI values by sex and genotype in *Ercc1*^−/Δ^ mice vs. wt. Significant effect of genotype on CFI value (two-way ANOVA; *F*(1, 12) = 196.50, *p* < .001), but no significant effect of sex (two-way ANOVA; *F*(1, 12) = 1.05, *p* = .325) or genotype × sex interaction (two-way ANOVA; *F*(1, 12) = 3.75, *p* = .077). **E** Mean $\pm$ SEM comparison of CFI scores by age and genotype in *Xpg*^−*/*−^ mice vs. wt. It should be noted that one male *Xpg*^−*/*−^ mouse died between mSFI and CFI testing, leaving *N* = 7. Significant effect of genotype on CFI value (two-way ANOVA; *F*(1, 24) = 248.00, *p* < .001), but no effect of sex (two-way ANOVA; *F*(1, 24) = 0.008, *p* = .931) or genotype × sex interaction (two-way ANOVA; *F*(1, 24) = 0.004, *p* = .951). *Significant difference between groups from Šidák’s multiple comparisons test. Two symbols: *p* < .01; four symbols: *p* < .0001 post hoc

Next, we aimed to further validate the mSFI as a measure of biological aging beyond the characterization of chronological age by examining its validity with *bonafide* models of segmental progeria syndromes. As the mSFI is designed to measure social behavioral deficits that occur as the result of the biological aging process, we hypothesized that the accelerated biological aging characteristic of progeroid syndromes would lead to greater social frailty compared to wild-type controls. We tested this hypothesis in two separate well-characterized mouse models of progeria: *Ercc1*^−/Δ^ and *Xpg*^−*/*−^ mice [64, 69]. *Ercc1*^−/Δ^ and *Xpg*^−*/*−^ mice model distinct human segmental progeria syndromes, but both involve the accumulation of DNA damage due to deficits in DNA repair [64, 66, 69, 98] (Fig. 4A).

As *Ercc1*^−/Δ^ and *Xpg*^−*/*−^ mice are both produced on a hybrid C57BL6/FVB background, hybrid C57BL6/FVB background mice served as wild type (wt) and were used as sex-matched reference samples from which to calculate mSFI values. To avoid confounds in wt mice caused by differences in breeding, each model was compared to respective wt littermates. The exact reference values that were obtained in each item from *ERCC1* wt and *XPG* wt mice can be found in Supplementary Table 3. Both the mSFI and CFI were applied in male (*N* = 4) and female (*N* = 4) *Ercc1*^−/Δ^ mice as well as male (*N* = 4) and female (*N* = 4) *ERCC1* wt mice at 8 weeks of age to anticipate the rapid physical decline observed in these progeria models (Fig. 4B, D; Supplementary Fig. 1C).

*Ercc1*^−/Δ^ mice exhibited significantly elevated mSFI values compared to *Ercc1* wt (Fig. 4B). Interestingly, a significant interaction between genotype × sex was found, which manifested in a significant elevation in male *Ercc1*^−/Δ^ mSFI values compared to female *Ercc1*^−/Δ^ mice. For reference, male *Ercc1*^−/Δ^ mice exhibited mSFI values like those observed in the oldest group-housed C57BL/6 mice tested (*Ercc1*^−/Δ^ = 0.40 $\pm$ 0.02; 34-month-old C57BL/6 = 0.39 $\pm$ 0.04) whereas female *Ercc1*^−/Δ^ mice exhibited greater mSFI values compared to the oldest group-housed C57BL/6 mice tested (*Ercc1*^−/Δ^ = 0.29 $\pm$ 0.02; 34-month-old C57BL/6 = 0.20 $\pm$ 0.03). As expected, based on the multi-system physiological consequences of their progeria phenotype, *Ercc1*^−/Δ^ mice showed parallel significant increases in CFI values compared to *Ercc1* wt (Fig. 4D). However, no effect of sex or genotype × sex interaction on CFI values was found. Male *Ercc1*^−/Δ^ mice exhibited CFI values higher than those observed in the oldest group-housed C57BL/6 males tested (*Ercc1*^−/Δ^ = 0.13 $\pm$ 0.01; 34-month-old C57BL/6 = 0.11 $\pm$ 0.02); female *Ercc1*^−/Δ^ CFI values were similar to that observed at 21 months of age in group-housed C57BL/6 females (*Ercc1*^−/Δ^ = 0.16 $\pm$ 0.01; 21-month-old. C57BL/6 = 0.16 $\pm$ 0.01).

Moreover, male (*N* = 8) and female (*N* = 6) *Xpg*^−*/*−^ mice were administered the mSFI and CFI at 8 weeks of age along with male (*N* = 8) and female (*N* = 7) wt mice (Fig. 4C, E; Supplementary Fig. 1D). *Xpg*^−*/*−^ mice exhibited significantly elevated mSFI values compared to *Xpg* wt mice; however, this effect occurred independent of sex (Fig. 4C). Male *Xpg*^−*/*−^ mice had mSFI values (0.24 $\pm$ 0.02) comparable to those observed in C57BL/6 males aged 16 to 21 (*XPG*^−*/*−^  = 0.24 $\pm$ 0.02; 16-month-old C57BL/6 = 0.20 $\pm$ 0.02; 21-month-old C57BL/6 = 0.29 $\pm$ 0.02); female *Xpg*^−*/*−^ mice (0.29 $\pm$ 0.02) showed greater mSFI values than observed in the oldest age group of C57BL/6 females tested in our study (*Xpg*^−*/*−^  = 0.29 $\pm$ 0.02; 34-month-old C57BL/6 = 0.20 $\pm$ 0.03). Also expected due to the documented severity of their progeroid syndrome, *Xpg*^−*/*−^ mice exhibited significantly elevated CFI values compared to wt independent of sex (Fig. 4E). Male *Xpg*^−*/*−^ mice exhibited CFI scores as high as those observed in the oldest group-housed C57BL/6 males tested (*Xpg*^−*/*−^  = 0.13 $\pm$ 0.01; 34-month-old C57BL/6 = 0.11 $\pm$ 0.02), whereas female *Xpg*^−*/*−^ CFI scores were between that observed at 16 and 21 months of age in group-housed C57BL/6 females (*Xpg*^−*/*−^  = 0.13 $\pm$ 0.01; 16-month-old C57BL/6 = 0.06 $\pm$ 0.01; 21-month-old C57BL/6 = 0.16 $\pm$ 0.01).

The observed significant increases in social frailty in both *Ercc1*^−/Δ^ and *Xpg*^−*/*−^ mice compared to age-matched wt mice prove that the mSFI is sensitive to genetically induced accelerations of the biological aging process.

## Discussion

### Development and validation of a novel index of social frailty in mice

Here, we present the development of the first index of social frailty in mice, and its initial validation in naturally aged group-housed male and female C57BL/6 mice in addition to models in which biological aging is accelerated genetically (progeria) or environmentally (social stress, individual housing).

The mSFI is based on the deficit-accumulation model of frailty and optimized for use in mouse longitudinal studies. The mSFI is relatively simple to execute and minimally invasive in measuring essential facets of social behavioral functioning that might become impaired during the aging process in both sexes of mice. Social/functional impairment may manifest at different levels and facets of social capabilities within individual mice making the application of a single social behavioral assay inadequate to capture age-related changes. Therefore, the mSFI consists of multiple well-established, high-throughput social behavioral assays. Although the inclusion of numerous items in frailty indices (e.g., 31 items characterize the CFI) has been shown to allow greater discrimination between graded levels of age-related impairment in both mice and humans, only seven assays were chosen to enable the large-scale application of the mSFI while minimizing the behavioral and physiological impact of more extensive procedures [22, 23, 29, 31].

Chronological age-dependent increase mSFI scores observed in both male and female C57BL/6 group-housed mice from the commonly used NIA aged rodent colony provide strong support for the construct validity of the mSFI as a measure of functional impairment in the social domain that accumulates during the aging process. The validity of the mSFI is also supported by multiple other lines of evidence, including (i) the relationship between the mSFI and the CFI, which is the golden standard assessment of physical frailty in mice; (ii) increased mSFI values in experimental social manipulation protocols known to elicit a chronic stress response; and (iii) increased mSFI values in progeria models.

While the correlation between the mSFI and the CFI provides support for the criterion validity of the mSFI, the lack of linearity in their relationship serves to distinguish the two measures. This further suggests that it may be possible to differentiate two independent dimensions of frailty during aging, across sexes. Specifically, our results in group-housed C57BL/6 mice revealed that males accumulate social impairments at earlier ages in the lifespan and at much quicker rates in relation to their level of physical impairments. Conversely, females exhibited a delayed accumulation of social impairments despite accruing a greater magnitude of physical impairment with age [93]. This intrinsic difference is highlighted by the opposing concavity of the best-fit quadratic describing the relationship between mSFI and CFI in each sex. These results suggest that male mice may manifest early age-related impairment in the social domain, whereas females may primarily manifest physical impairment with age while remaining socially apt.

Importantly, social frailty only manifests at a very old age in group-housed C57BL/6 females. Different factors can be invoked to explain this finding.

First, while relevant for mouse biology, the items used to calculate the mSFI may capture socio-behavioral phenotypes that are more salient to males than females. While a future attempt can be to refine a female-specific index, at least three results seem to exclude a strong sex-specific bias in the items used to calculate the mSFI: (i) the relative contribution across items in the reference 4-month-old group is similar between males and females; (ii) both *Ercc1*^−/Δ^ and *Xpg*^−*/*−^ mice manifest a sex-independent increase in mSFI compared to wild types; (iii) male *Ercc1*^−/Δ^ show higher mSFI values compared to female *Ercc1*^−/Δ^ mice.

Second, a larger variability is noticeable in females across most all age groups. While the source of this variability is currently unknown, low variability in the reference group is critically relevant for the calculation of deficit-accumulation frailty indexes [29]. Thus, future work may optimize the age/condition for the calculation of a reference group with limited intrinsic variability in the mSFI score in females.

Third, sibling group housing is regarded as a minimally stressful housing condition in female mice, where they are allowed to exhibit natural social interaction, communication, and communal nesting [59, 88, 99–101]. Female mice are more gregarious, have less linear and despotic social hierarchies, and tend to maintain familiar social bonds more than males who generally disperse after sexual maturity and maintain stronger social divisions based on aggressive agonistic interactions [102–106]. In this light, our results suggest that the maintenance of a low level of social frailty with increasing age compared to males—in spite of increased level of physical frailty—may lie in socio-behavioral factors buffering the advent of social impairment in female mice.

### Social factors affect social frailty in male mice

Sibling group housing is also generally considered a less stressful housing condition in male mice [88, 99, 100], when compared to chronic social isolation [62, 107], and chronic social subordination [42, 56, 57]. Furthermore, the CSS method used in our study was developed as a model exacerbating age-related declines, shortening lifespan, and several hallmarks of aging in the increased rate of biological aging [42, 56, 57]. Based on this socio-behavioral evidence, and the prevailing framework conceptualizing social frailty [47], one would posit that mice lacking the opportunity to exhibit social skills (isolated individual housing) or challenged with an adverse social experience (psychosocial stress via submissive social status) would exhibit greater social frailty compared to mice with constant social stimulation (group housing). Our results are consistent with this hypothesis in showing that group-housed mice showed lower levels of social frailty than age-matched chronically isolated and chronically subordinate mice.

### The social frailty index increases in progeroid mouse models

The *Ercc1*^−/Δ^ and *Xpg*^−*/*−^ mice used in this study both present multisystem progeria phenotypes that compromise longevity and involve the early onset of complex age-related pathologies characteristic of accelerated biological aging [64–67].

The direct link we present between increased levels of both physical and social frailty in mouse models of genomic instability supports on the one hand the validity of the mSFI as a test capable to detect accelerated biological aging, and on the other hand, it suggests that it can be a useful tool to examine the impact of additional hallmarks of aging [108] on social functional impairment during aging. The clinical and social frailty impairments in 8-week-old *Ercc1*^−/Δ^ and *Xpg*^−*/*−^ mice match those of 16 + -month-old C57BL/6 mice could also be interpreted as the consequence of developmental defects induced in these models of human segmental progeria that affects multiple organ functions [67, 109, 110]. Future studies should focus on longitudinal testing along the entire (albeit short) lifespan to disentangle the role of developmental defects vs. accelerated aging in these progeria models.

### Translational relevance of the mSFI

Aging is characterized by multiple functional impairments, including a reduction of social interaction and social functioning. The growing interest towards social frailty in humans [47] has culminated in the recent development and application of a human Social Frailty Index by Shah et al. (2023) based on a cohort of 8250 adults aged 65 years and older. This index includes age, gender, and eight measures spanning general resources, social resources, social activities, and fulfillment of basic social needs [52]. Most items are based on parameters gleaned from self-report and observational data. Thus, the index measures categorical social outcomes that are consequential of social behavior but not social behavior itself. Importantly, the human Social Frailty Index was found to be a robust predictor of 4-year mortality.

Our mSFI quantifies real-time behaviors spanning similar categories quantified by Shah et al.’s (2023) Social Frailty Index: (i) general resources and social resources are controlled for by standard housing conditions; (ii) social interaction and social communication assays evaluate social activities; (iii) and fulfillment of basic social needs is evaluated in the nest building test. Although our index was not strictly derived to match the human index, in the context of our study the impairment in social capabilities that accompanies aging appears to be evolutionarily conserved and measurable across mice and humans, suggesting that the mSFI may be a translatable index useful for preclinical studies of the aging process. Future studies should attempt to explore the translatability of a general Social Frailty Index across species, as done by Whitehead et al. (2014) for the CFI.

In humans, a sex-based paradox exists between health, frailty, and survival. Women exhibit greater degrees of frailty and poorer health overall than their age-matched male counterparts despite experiencing lower mortality rates [111, 112]. Sociobehavioral explanations for this paradox have been posited in addition to biological ones [113, 114]. Work in humans examining links between frailty and “social vulnerability,” an alternative deficit accumulation-based conceptualization of the social manifestations of the aging process, found a positive relationship between frailty and social vulnerability but did not explicitly delineate by sex [51]. Other research in humans found that the correlation between frailty and social vulnerability was higher in women than men [46]. These contrasting findings highlight the lack of standardization and consistent measurement techniques in humans to quantify the social dimensions of frailty, warranting further investigation of the link between the two domains of frailty and the sex-based mediation of their relationship. Our finding that group-housed female C57BL/6 mice show *higher* levels of physical frailty compared to age-matched males is consistent with previous literature, but also in general accordance with the observed paradox in humans [93]. In this light, our contrasting finding that the same female C57BL/6 mice exhibited *lower* social frailty levels than age-matched males may have very important implications for discriminating different aging trajectories between the sexes.

Overall, the mSFI provides a new translationally relevant tool to define and investigate the mechanisms driving functional impairments during the aging process.

## Conclusion

A strength of our study lies in comparisons of social frailty using the mSFI over a wide range of ages across the lifespan of male and female laboratory mice, as well as its validation in progeroid strains and in male mice exposed to two independent social manipulations. However, some limitations must be considered. Given the age-related increase of mortality, there were small sample sizes in older age groups creating low-powered comparisons therein. Also, this study primarily focused on applying mSFI cross-sectionally rather than longitudinally and contains no association of social frailty with mortality or pathological outcomes at the individual level. A longitudinal study that repeatedly applies the mSFI to both wild-type mice and progeria models during the entire lifespan that spans as close as possible to natural death is necessary to characterize the validity of this index to predict disease onset and age at death. The lack of longitudinal data and our exclusive use of males also hinders our interpretation of the effect of individual housing and psychosocial stress on social frailty. Similarly, a major future direction would be to apply the mSFI to intervention studies designed to improve healthspan and/or extend lifespan. Lastly, the mSFI was developed to measure social behavior specifically in mice. In identifying key experimental variables amenable to quantitative analysis of social behavior, our study suggests approaches to develop indexes of social frailty that can be valid across context and species, likely requiring modification of the individual items to reflect the species-specific social repertoire.

In conclusion, we developed and initially validated a new index for social frailty in mice. The mSFI detects a progressive age-dependent increase of social frailty through the lifespan in group-housed mice, with female mice exhibiting a delayed onset, and lower level of social frailty compared to males. Further, we demonstrated that the mSFI is sensitive to experimental manipulations known to accelerate biological aging such as individual housing, social stress, and progeroid syndromes [42].

Overall, the mSFI represents a new approach to quantify a previously untapped dimension of functional impairment in mouse models of aging. We postulate that its use in conjunction with other indexes of functional deficit [29, 115], and measures of biological age [108], may help refine our understanding of various dimensions of the aging process.

## Supplementary information

Below is the link to the electronic supplementary material.

Supplementary file1 (DOCX 273 KB)

Supplementary file2 (DOCX 144 KB)

Supplementary file3 (DOCX 55 KB)

## Data Availability

Data will be made available upon request to the corresponding author.
