# Supplementary material for: The mouse Social Frailty Index (mSFI): a novel behavioral assessment for impaired social functioning in aging mice
Source: GeroScience. 2024 Jul 11;47(1):85–107. doi: 10.1007/s11357-024-01263-4 (PMC11872866; doi:10.1007/s11357-024-01263-4)
Supplement: Supplementary file 1 — Supplementary file1 (DOCX 273 KB) [file 11357_2024_1263_MOESM1_ESM.docx]

**The mouse Social Frailty Index (mSFI) Methodology**

**Bartolomucci Lab | University of Minnesota**

The mouse Social Frailty Index (mSFI) was designed to quantify age-related impairments of social functioning in mice as well as to identify experimental manipulations accelerating or delaying the age-related onset of social impairment.

The mSFI consists of a set of seven minimally invasive behavioral assays that assess areas of social behavioral functioning in mice including social communication, social interaction, and social functional ability. The following document outlines the standard operating procedure for how to perform and calculate the mSFI.

**Narrative description of the mSFI**

The seven social behavioral assays that comprise the mSFI are the olfactory test, urine marking test, urine countermarking test, home cage social interaction test, novel cage social interaction test, social approach/avoidance test, and nest building test. The olfactory test, urine marking test, and urine countermarking test are assays of social communication skills. The home cage social interaction test, novel cage social interaction test, and social/novel object preferencetest are assays of social interaction skills. Lastly, the nest building test is an assay of social functional ability.

Provided the availability of suitable working surfaces (tables, shelves, carts, etc.), the mSFI can be conducted in the same room where the experimental mice are housed.

Assays in the mSFI occur in both the home cage environment (olfactory test, social interaction in home environment, nest building) and novel cage environments (urine marking, urine countermarking, social interaction in a novel environment, social/novel object preference test and nest building – see individual sections for specifics). In the case where mice are not individually housed, special consideration should be taken for assays that occur in the home cage environment. Mice housed in groups should be assigned to undergo testing in the home cage environment in random order. During testing, group-housed cage mates to be tested subsequently can be housed in temporary cages. When this occurs, the group-housed experimental mice that are undergoing testing should be allowed to freely roam the home cage for one minute to habituate to the novel conditions before behavioral testing occurs. For home cage testing in CSS-housed mice (Bartolomucci et al., 2001), CD1 mice should be removed from the cage and temporarily housed while maintaining the divider in place. The experimental mouse should then be allowed to habituate to the novel conditions before testing. At the end of the testing period, the CD1 mouse can be promptly reintroduced to the shared home-cage. The mSFI can be applied in most housing conditions insofar as it is a true home environment, the experimental mouse is isolated for testing, and a proper behavioral testing environment is maintained (no loud noises, scents, sudden movements, etc.).

Each of the following seven procedures should be repeated on each experimental animal to completion and until all desired experimental animals are tested. The order of application on experimental mice in each assay should be randomized.

**Procedures**

The seven social behavioral assays that comprise the mSFI are designed to be conducted during AM and PM sessions over 3.5 days.

***Item #1. Olfactory Test – Home Environment***

This assay evaluates the ability of mice to spontaneously discriminate between social odors and nonsocial odors and is a procedural modification of the olfactory habituation/dishabituation test (Moreno et al., 2014; Witt et al., 2009).

Materials

1. 15.24 cm Cotton swabs (e.g., Tyco Healthcare Group, LP, Mansfield, MA; Kendall Curity™ Single Tipped Applicators REF no. 8884540500).
2. Tap water.
3. 1:10,000 pure vanilla extract (McCorrmick & Co., Inc., Hunt Valley, MD) diluted in tap water.
4. Pooled male + female urine (Biochemed Pharmacologicals, Winchester, VA; amount ~2mL/20 mice).
5. 1 Stopwatch per experimenter + 1 additional to keep track of sniffing intervals.

Procedure

1. Place the experimental mouse home-cage on the working surface (see above), remove the cage filter top (if present), and while maintaining the metal lid and food in place set the water bottle aside.
2. For each of the three odors - water, vanilla, urine, in that order - the process is identical:
   1. Wet the cotton swab in stimulus odor solution, making sure to press the cotton tip firmly against the edge of the tube to get rid of excess liquid.
   2. Ready stopwatch with preferred hand. This will be used to measure time spent sniffing the swab.
   3. Ready an extra timer that will be used for 2-minute interval timing.
   4. With cotton swab preferred hand, insert swab approximately 2.5 cm through wire top near water bottle insert, starting 2-minute interval timer simultaneously.
   5. Observe the mouse behavior intently for the duration of the 2 minutes. Starting the stopwatch only when the mouse is engaging with the cotton swab (see below) and stopping it as immediately as possible when the mouse disengages.
      1. Engaging behavior is defined as: the mouse nose is within approximately 2 cm of the cotton swab, oriented towards the swab, and making visible efforts to inhale the odor (i.e. the mouse is actively sniffing the swab, not simply walking past it); the mouse is chewing, licking and/or gnawing at the swab; the mouse is holding the swab with its forepaws).
   6. After each 2-minute interval, remove the cotton swab and record the time spent sniffing the odor.
   7. Allow a 1 min recovery period before presenting the next odor.

Note: This is the longest assay in the mSFI. At maximum efficiency, it takes 8 minutes per mouse. With two experienced experimenters it takes approximately 2 hours to test 20 mice.

*Olfactory Test Quantification:* The statistic of interest in the Olfactory Test is the percentage of social odor preference or the % urine (social odor) preference.

Formula: *% urine preference = time spent sniffing urine (s) / total time spent sniffing odors (s) x 100*.

***Item #2. Urine Marking – Novel Environment***

This assay evaluates the ability of mice to socially communicate via strategically placing urinary marks in a novel environment.

Materials

1. Empty (i.e., no bedding, nesting material, food, and water bottle) small static cages (186 mm x 298 mm x 128 mm).
2. 31.5 x 33.5 cm Whatman 3MM CHR chromatography blotting paper cut to size of test cages (Cytiva, Marlborough, MA; CAT no. 3030-335).

Procedure

1. Before the start of testing, cut Whatman paper into sheets according to the standard size of cages used in your animal care facility so that the paper will lay flat and cover the entire bottom of the cage during experimentation. Label with any pertinent identification information (i.e., date, subject ID, etc.).
2. Lay labeled sheets of Whatman paper within experimental cages, ensuring that the paper lies flat on the bottom of the cage.
3. Place experimental mice in cages with Whatman paper and close the cages.
4. Once they are all in their respective cages, allow mice to freely roam the novel environment and deposit their urinary marks for 1 hour.
5. At the end of the 1-hour period, return the mice to their home-cages.
6. Remove the marked Whatman paper, clean any feces, and hang to dry overnight to allow for the fixation of urinary marks.

Note: The urine marking assay in its entirety with two experimenters took approximately 1.25 hours per 20 mice. This does not include quantification which is highly variable depending on experience.

*Details on quantification and analysis can be found after #3*.

***Item #3. Urine Countermarking – Novel Environment***

This assay evaluates the ability of mice to respond to social communication in the form of conspecific urinary marking via strategically placing their urinary countermarks around a novel environment.

Materials

1. Empty (i.e., no bedding, nesting material, food, and water bottle) small static cages (186 mm x 298 mm x 128 mm).
2. 31.5 x 33.5 cm Whatman 3MM CHR chromatography blotting paper cut to size of test cages (Cytiva, Marlborough, MA; CAT no. 3030-335).
3. Pooled male + female urine (Biochemed Pharmacologicals, Winchester, VA; amount 0.2mL/20 mice).

Procedure

1. Before the start of testing, cut Whatman paper into sheets according to the standard size of cages used in your animal care facility so that the paper will lay flat and cover the entire bottom of the cage during experimentation. Label with any pertinent identification information (i.e., date, subject ID, etc.).
2. Using a pencil outline a small circle in the center of the Whatman paper, this will serve as a reference for where to aliquot the stimulus urine immediately before testing.
3. Prepare empty novel cages with labeled sheets of Whatman paper, ensuring that the paper lies flat on the bottom of the cage.
4. Aliquot 10 µL of the pooled urine into the centrally located reference outline. This will serve as the stimulus for countermarking behavior.
5. Immediately place experimental mice in cages with Whatman paper and close the cages.
6. Once they are all in their respective cages, allow mice to freely roam the novel environment and deposit their urinary marks for 1 hour.
7. At the end of the 1-hour period, return the mice to their home-cages.
8. Remove the marked Whatman paper, clean any feces, and hang to dry overnight to allow for the fixation of urinary marks.

Note: The urine countermarking assay in its entirety with two experimenters took approximately 1.25 hours per 20 mice. This time does not include quantification which is highly variable depending on experience.

*Urine Marking & Countermarking Quantification and Analysis*

Materials

1. UV transilluminator (e.g., Ultra-Violet Products, Inc., San Gabriel, CA; Chromato-Vue Transilluminator Model 0 – 62).
2. High-resolution camera – high resolution phone camera is also ok.
3. ImageJ (Version 1.53k; Wayne Rasband & NIH, Bethesda, MD)

*Before conducting the following procedure, ensure that the marked Whatman paper is completely dry.

UV Scanning

1. Turn off the lights in the room.
2. Place marked Whatman 3MM Chromatography paper as flat as possible on a large UV transilluminator.
   1. The paper should be completely in the illuminated area.
   2. Streaking due to UV light placement should not align with the edges of the paper when in view of the camera.
3. Using any standard high-resolution camera, take a picture of the paper.
   1. Before taking the picture, ensure that measures are taken to ensure that the photo is saved as a jpeg.

Example Quantification using ImageJ Software

1. Import images.
2. Drag and drop the image into open ImageJ window.
3. Left-click on the rectangle tool to select the ‘rotated rectangle’ tool.
   1. Using the rotated rectangle tool, outline the paper.
4. Select ‘Image’, ‘Type’, and ‘8-bit’ to convert to grayscale.
5. Under ‘Image’ select ‘Threshold’
   1. ‘Default’ and ‘B&W’ should be displayed.
   2. Move the bottom slider to ‘255’
   3. Utilize the top slider to threshold the image so that urine-marked spots within the paper are black in color on the white background of the paper. Exact B&W values will vary depending on the picture.
6. Once the optimal threshold is achieved, select ‘Analyze’ and ‘Analyze Particles’.
   1. Under ‘Size (pixel^2)’ enter 100 as a minimum limit and 40000 as a maximum to ensure that no ‘noise’ from lighting is detected and that no large urine spots are detected – these are not a display of marking behavior but rather pure urination.
   2. Check ‘Display results’, ‘Overlay’, and ‘Summarize’.
   3. Select ‘OK’.
   4. A summary window will pop up displaying some values, the only one that pertains to behavioral quantification in these two assays are ‘count’ which is the number of markings present, the statistic of interest for both assays.
      1. Note for countermarking: ‘Count’ will display one more mark than is actually there due to the 10µL of pooled urine added as a stimulus. You will need to subtract this single mark from the number of markings reported.

Formulas:

1. Urine Marking:  *Number of markings = Number of markings*
2. Urine Countermarking: *Number of markings = Number of markings - 1*

***Item #4. Social Interaction Test – Home Environment***

Introduction of a same-sex juvenile conspecific to the home environment elicits a variety of social behaviors without many of the confounds of dominant aggression, thus this assay evaluates and quantifies sociability. The following protocol is a modification of the resident-intruder test (Gascon et al., 2014; Winslow, 2003).

Materials

1. Stopwatch.
2. Sex- and strain-matched juvenile (4-5 weeks old) stimulus mice – testing can be conducted with up to 5 mice per experimenter. Juvenile mice can be reused in subsequent rounds, so the amount required is one that is sufficient to provide one round of experimental mice with sex-matched juveniles (i.e., if you have 20 mice, 10 of which are males and 10 of which are females, you would only need 5 male and 5 female stimulus mice which would be used in two rounds of experimentation). If juvenile mice are of the same coat color as the experimental mouse, mark the tail of the juveniles with a bright-colored marker for ease of observation and to differentiate between juveniles themselves.
3. Coding sheet for instantaneous sampling (see example in appendix).

Procedure

1. Place the home cages on the working surface where the observations will be conducted (up to 5 cages per observer), aligning them with the long sides of the cages facing the centrally located experimenter.
2. Place stimulus juvenile mice in the home environment of the experimental mice, closing the cage afterward.
   1. Note: if all stimulus mice cannot be placed in experimental home environments simultaneously, place female stimulus mice in first and male stimulus mice in second. This will minimize the time in which aggressive behavior by the male mice might manifest before the observation period begins.
3. Allow mice to freely roam the cage for 5 minutes before ending instantaneous observation and removing the stimulus mouse. The procedure for instantaneous sampling during and behavioral characterizations are described after #5.
4. In the circumstance that aggressive behavior between experimental and stimulus mice escalates to the point where there is a threat of wounding for either mouse, the interaction should be immediately terminated.

Note: The social interaction test in the home environment in its entirety with two experimenters took approximately 45 minutes per 20 mice.

Analysis is described after #5.

***Item #5. Juvenile Social Interaction – Novel Environment***

In the presence of both a novel same-sex juvenile and a novel environment, a conflict arises between social motivations to investigate a social stimulus and motivations to investigate a novel environment. This assay evaluates the level of social inclination a mouse has in the presence of many stimuli that provoke evolutionarily advantageous behaviors. This protocol is identical to the juvenile social interaction test in the home environment, just in a novel environment.

Materials

1. Stopwatch.
2. Static cages with a layer of bedding but without nesting material – for ease of observation.
3. Sex- and strain-matched juvenile (4-5 weeks old) stimulus mice – testing can be conducted with up to 5 mice per experimenter. Juvenile mice can be reused in subsequent rounds, so the amount required is one that is sufficient to provide one round of experimental mice with sex-matched juveniles (i.e., if you have 20 mice, 10 of which are males and 10 of which are females, you would only need 5 male and 5 female stimulus mice which would be used in two rounds of experimentation). If juvenile mice are of the same coat color as the experimental mouse, mark the tail of the juveniles with a bright-colored marker for ease of observation and to differentiate between juveniles themselves.
4. Coding sheet for instantaneous sampling (see example in appendix).

Procedure

1. On the working surface, align up to 5 novel static cages in a semi-circle with the long sides of the cages facing the centrally located experimenter.
2. Place stimulus juvenile mice in the novel environment and close the cage.
3. Remove experimental mice from their home cage and place them into the novel environment with the stimulus juvenile mice.
   1. Note: Experimental mice must be placed into the novel environment last to ensure that ALL exploratory behavior that is observed is done in the presence of the stimulus juvenile mouse.
4. Allow mice to freely roam the cage for 5 minutes before ending instantaneous observation and removing the stimulus mouse. See quantification and analysis section for specifics on instantaneous observation.
5. In the circumstance that aggressive behavior between experimental and stimulus mice escalates to the point where there is a threat of wounding for either mouse, the interaction should be immediately terminated.

Note: *Experimental mice should never be exposed to the same juvenile twice to prevent social recognition and decreased interaction (Bluthe et al., 1993; Dantzer, 1999).* The social interaction test in the novel environment in its entirety with two experimenters took approximately 45 minutes per 20 mice.

*Home & Novel Environment Juvenile Social Interaction Test Quantification and Analysis*

Observation and Instantaneous Sampling: During the 5-minute interaction, experimental mice are observed for 1 second sequentially (i.e., each mouse is observed every 5 seconds), and instances of social behavior initiated by the experimental mouse are recorded. The experimenter should scan left to right along the semi-circle of cages, spending 1 second observing each mouse’s behavior while simultaneously recording behavior. Social behaviors measured in this test span categories of sniffing, aggression, and allo-grooming and are displayed in the ethogram in Table 1 below.

| **Category** | **Behavior** | **Description** |
| --- | --- | --- |
| Sniffing | Pursuit | Closely follows and chases stimulus animal. |
|  | Olfactory investigation | Uses nose to inspect any portion of the stimulus mouse’s body, including the tail. Initially, the area of interest is typically the anogenital region of the stimulus animal. |
| Aggression | Attack bite | Bites are usually focused on the rump or hind of the stimulus mouse, typically associated with an escape/leap. |
|  | Sideways offensive posture | Subject mouse positions its body perpendicular to the front of the stimulus animal. While having its side facing the head of the stimulus mouse, the subject mouse orients its head towards the stimulus animal's hindquarters. The body of the subject mouse may appear U-shaped. Must occur with a physical attack. |
|  | Mount | Appears to attempt to copulate with the stimulus animal. The observed animal may climb and perform pelvic thrusts onto the rump, side, or head of the stimulus animal. |
| Allo-grooming | Allo-grooming | Licks, cleans, and/or physically probes the fur of the stimulus mouse. This usually occurs close to the facial, neck, or upper back regions. |

Table 1: Mouse social behavior ethogram for use in social interaction tests – adapted from Winslow (2003).

Formula: A % social interaction is calculated from the social interaction test in the home and in a novel environment in identical fashions: *% social interaction = (sniffing intervals + aggression intervals + allo-grooming intervals) / total # intervals observed x 100.*

Theoretically, the number of intervals scored over 5 minutes should be 60, however, this varies slightly between observers due to experience or general speed. If an interaction requires termination due to escalated aggressive behavior, the test should be scored as is (i.e. total # of intervals observed = how many intervals occurred before interaction termination.)

***Item #6. Social/Novel Object Preference Test – Novel Cage***

This assay evaluates social approach behavior, which in a neutral environment devoid of threats, can be used to measure the dynamics of sociability and anxiety. The following protocol is a modification of the three-chamber social approach task (Crawley, 2007; Yang et al., 2011).

Materials

1. Large static cages (257 mm x 483 mm x 152 mm) with a layer of bedding but without food, water, nesting material, or metal wire lids.
2. Sex- and strain-matched juvenile (4-5 weeks old) stimulus mice – testing is conducted in groups of five mice per experimenter. Juvenile mice can be ‘recycled’ in subsequent rounds, so the amount required is one that is sufficient to provide one round of experimental mice with sex-matched juveniles (i.e., if you have 20 mice, 10 of which are males and 10 of which are females, you would only need 5 male and 5 female stimulus mice which would be used in two rounds of experimentation).
3. Novel objects (Ex: Simport™ Scientific SecurTainer™ 40mL Tamper Evident Specimen Container; Simport Scientific Inc., Saint-Mathieu-de-Beloeil, Quebec). However, these can be changed based on available resources, they just have to fit in the wire mesh cups and be similar in size to a juvenile mouse.
4. 2 wire mesh cups per cage (Ex: Amazon Basics Wire Mesh Pen Cup, Amazon, Seattle, WA).
5. 2 bottles filled with tap water (Ex: Milipore Express® PLUS 0.22 mm PES 150mL bottle unit; EMD Milipore Corporation, Burlington, MA) or any other available object to serve as a weight on top of the wire mesh cups to prevent the mouse from tipping them while exploring.
6. Spray bottle containing 70% EtOH.
7. Coding sheet for instantaneous sampling (see example in appendix).

Procedure

1. In the experimental area align up to 5 novel large static cages in a semi-circle with the long sides of the cages facing the centrally located experimenter.
2. To assemble the social/novel object preference apparatus in each cage, place a wire mesh cup containing the stimulus same-sex juvenile on one side of the cage and a wire mesh cup containing the novel object on the side opposite.
   1. The side on which each is placed should alternate in subsequent cages.
   2. The cups should be far enough from each other so that the experimental mouse has to *choose* to approach them (i.e. not sit in the middle and have access to both) while still allowing enough room for the mouse to maneuver behind it.
3. Place the cup or other container filled with water on each wire mesh cup.
4. Place experimental mice into the assembled cages and close the filter top.
5. Allow mice to freely roam the cage for 5 minutes before ending instantaneous observation and removing the experimental mouse. See quantification and analysis section for specifics on instantaneous observation.
6. If doing another round of testing: after removing the experimental mice, remove the stimulus mice, then remove the objects, wire mesh cups, and water-filled containers. Clean the objects, wire mesh cups, and water-filled containers with 70% ethanol and allow them to dry for 1 minute before reusing them.

Note: *Experimental mice should never be exposed to the same juvenile twice to prevent social recognition and decreased interaction (Bluthe et al., 1993; Dantzer, 1999).*

The social/novel object preference test test in its entirety with two experimenters took approximately 1.25 hours per 20 mice.

*Social/Novel Object Preference Quantification and Analysis*

Observation and Instantaneous Sampling: The instantaneous sampling method used for this test is similar to those described in the juvenile social interaction tests, however, the behaviors recorded are different. Each experimental mouse should be observed for 1 second, every 5 seconds (if 5 mice are being tested at a time) via the aforementioned scanning method. Behaviors measured in the social/novel object preference test are simply interactions with the stimulus mouse and interactions with the object. Experimental mice are considered to be interacting with either the stimulus mouse or the object when actively sniffing, or digging around the wire mesh cup, but also when they are oriented towards it (i.e. stretch attend posture, rearing or climbing on it) in close proximity to the wire mesh cup.

Formula: *% social approach = (# intervals spent interacting with mouse - # intervals spent interacting with object) / total # spent interacting x 100.* Theoretically, the number of intervals scored over five minutes should be 60, however, again, this varies slightly between observers due to experience or general speed.

***Item #7. Nest Building – Home Environment***

This assay evaluates innate, goal-directed nest building behavior as a proxy for social functional ability. The following protocol is adapted from Deacon (2006; 2012).

Materials

1. Standard pressed cotton batting nestlets (Ancare Corp, Bellmore, NY).

Procedure

1. Approximately 1 hour before the dark phase remove all nesting material from the experimental mouse home cage.
   1. If testing mice that are group-housed, experimental mice are transferred along with bedding from their home cage into novel standard cages without nesting material (to be returned to their home cage after assessment).
2. Place one new intact cotton batting nestlet in the mouse cage and allow mice to build their tests overnight.
3. In the subsequent A.M. session, making sure not to disturb the natural structure of the built nest, assess the nest visually while the mouse is still in the cage using the published scale provided below (Deacon, 2006; 2012).

*Nest Building Quantification*

Nests are assessed visually, and subsequently rated based on the 5-point scale detailed in Table 2 below. Examples of what a nest of each score looks like have been described elsewhere (Deacon, 2006).

| **Nest Score** | **Criteria** |
| --- | --- |
| 1 | Nestlet not noticeably touched: more than 90% intact. |
| 2 | Nestlet partially torn: 50 – 90% intact. |
| 3 | Nestlet mostly shredded but no identifiable nest site present: 50 – 10% intact. |
| 4 | Mouse has constructed an identifiable but flat nest: nestlet more than 90% shredded. |
| 5 | A near perfect nest. More than 90% of the Nestlet is shredded and formed into a crater-like structure with walls higher than mouse body height. |

Table 2: Nest building test scoring criteria – adapted from Deacon (2006).

**mSFI Calculation**

Each assay produces a quantitative score that represents behavioral performance in its respective dimension of social behavior (e.g., a % urine preference is calculated from the Olfactory Test). Similar to the standard procedure in deficit accumulation-based frailty indices (Searle et al., 2008; Whitehead et al., 2014), an mSFI from 0 to 1 is calculated for each mouse based on the extent of deviation from mean quantitative scores obtained in an initial population of young adult mice (3 - 4 months of age) that undergoes mSFI testing Thus, before using this index in mice beyond four months of age, it first needs to be applied in a reference population of same-sex and strain young adult mice to obtain reference values. An mSFI of 0 represents an animal with no impairment in social functioning and an mSFI of 1 represents an animal with maximal impairment in social functioning. Between these minimal and maximal scores exists a gradient of impairment levels with greater values representing greater social frailty.

To calculate the comprehensive mSFI, quantitative scores in each item are assigned ‘index scores’ as follows: scores less than ±1 standard deviation (SD) of the reference population mean were assigned a 0, ≤ 2 SD = 0.25, ≤ 3 SD = 0.5, ≤ 4 SD = 0.75, > 4 SD = 1. It should be noted this methodology is not used in the case of the Nest Building assessment because it is scored on a 5-point scale. Instead, mSFI values were assigned so that nest scores of 5 received an index score of 0, 4 = 0.25, 3 = 0.5, 2 = 0.75, and 1 = 5. Index values for each mouse over all seven items are summed and divided by seven, the total number of items, to obtain a final mSFI value.

**Notes**

Typically, testing can start on a Monday and end on a Thursday, allowing Friday to be used for other testing such as the 31-Item Clinical Frailty Index assessment (Whitehead et al., 2014). The mSFI test sequence is meant to be flexible in the way that any test can be scheduled in any of the AM or PM sessions over the 3.5 days, thus compensating for in-house or outside scheduling demands without any impact on the outcome. The only exception is represented by nest building which needs to occur overnight. Special attention should be paid to when the AM and PM sessions occur each day. The AM sessions need to be scheduled early enough so that they do not go into the PM, but not too close to the start of the light cycle. The PM sessions need to be scheduled to give ample time between AM and PM sessions, but not too late that they come close to the start of the dark cycle. Typically, an AM start time of 8:30am works well and 1:30pm works well for the PM – besides the nest building test, see section for specifics.

**References**

Bartolomucci, A., Palanza, P., Gaspani, L., Limiroli, E., Panerai, A. E., Ceresini, G., Poli, M. D., & Parmigiani, S. (2001). Social status in mice: Behavioral, endocrine, and immune changes are context dependent. *Physiology & Behavior*, *73*(3), 401-410. <https://doi.org/10.1016/S0031-9384(01)00453-X>

Bluthe, R. M. Gheusi, G., & Dantzer, R. (1993). Gonadal steroids influence the involvement of arginine vasopressin in social recognition in mice. *Psychoneuroendocinology, 18*(4), 323-335. <https://doi.org/10.1016/0306-4530(93)90028-J>

Crawley, J. N. (2007). *What’s wrong with my mouse? Behavioral phenotyping for transgenic and knockout mice, 2^nd^ ed* (pp. xvi, 523). John Wiley & Sons Inc. <https://doi.org/10.1002/0470119055>

Dantzer, R. (1999). Vasopressin, gonadal steroids and social recognition. In I. J. A. Urban, J. P., H. Burbach, & D. De Wed (Eds.), *Progress in Brain Research* (Vol. 119, pp. 409-414). Elsevier. <https://doi.org/10.1016/S0079-6123(08)61584-8>

Deacon, R. (2012). Assessing burrowing, nest construction, and hoarding in mice. *JoVE, 59*, e2607. <https://doi.org/10.3791/2607>

Deacon, R. (2006). Assessing nest building in mice. *Nature Protocols, 1*(3), Article 3. <https://doi.org/10.1038/nprot.2006.170>

Gascon, E., Lynch, K., Ruan, H., Almeida, S., Verheyden, J. M., Seeley, W. W., Dickson, D. W., Petrucelli, L., Sun, D., Jiao, J., Zhou, H., Jakovcevski, M., Akbarian, S., Yao, W. D., & Gao, F. B. (2014). Alterations in microRNA-124 and AMPA receptors contribute to social behavioral deficits in frontotemporal dementia. *Nature Medicine*, *20*(12), Article 12. <https://doi.org/10.1038/nm.3717>

Moreno, M., Richard, M., Landrein, B., Sacquet, J., Didier, A., & Mandairon, N. (2014). Alteration of olfactory perceptual learning and its cellular basis in aged mice. *Neurobiology of Aging, 35*(3), 680-691. <https://doi.org/10.1016/j.neurobiolaging.2013.08.034>

Searle, S. D., Mitnitski, A., Gahbauer, E. A., Gill, T. M., & Rockwood, K. (2008). A standard procedure for creating a frailty index. *BMC Geriatrics, 8*(1), 24. <https://doi.org/10.1186/1471-2318-8-24>

Whitehead, J. C., Hildebrand, B. A., Sun, M., Rockwood, M. R., Rose, R. A., Rockwood, K., & Howlett, S. E. (2014). A clinical frailty index in aging mice: Comparisons with frailty index data in humans. *The Journals of Gerontology. Series A, Biological Sciences and Medical Sciences, 69*(6), 621-632. <https://doi.org/10.1093/gerona/glt136>

Winslow, J. T. (2003). Mouse social recognition and preference. *Current Protocols in Neuroscience, 22*(1), 8.16.1-8.16.16. <https://doi.org/10.1002/0471142301.ns0816s22>

Witt, R. M., Galligan, M. M., Despinoy, J. R., & Segal, R. (2009). Olfactory behavioral testing in the adult mouse. *JoVE, 23*, 949. <https://doi.org/10.3791/949>

Yang, M., Silverman, J. L., & Crawley, J. N. (2011). Automated three-chambered social approach task for mice. *Current Protocols in Neuroscience, 56*(1), 8.26.1-8.26.16. <https://doi.org/10.1002/0471142301.ns082s56>

**Appendix**

*Behavioral coding sheet for instantaneous sampling*

*
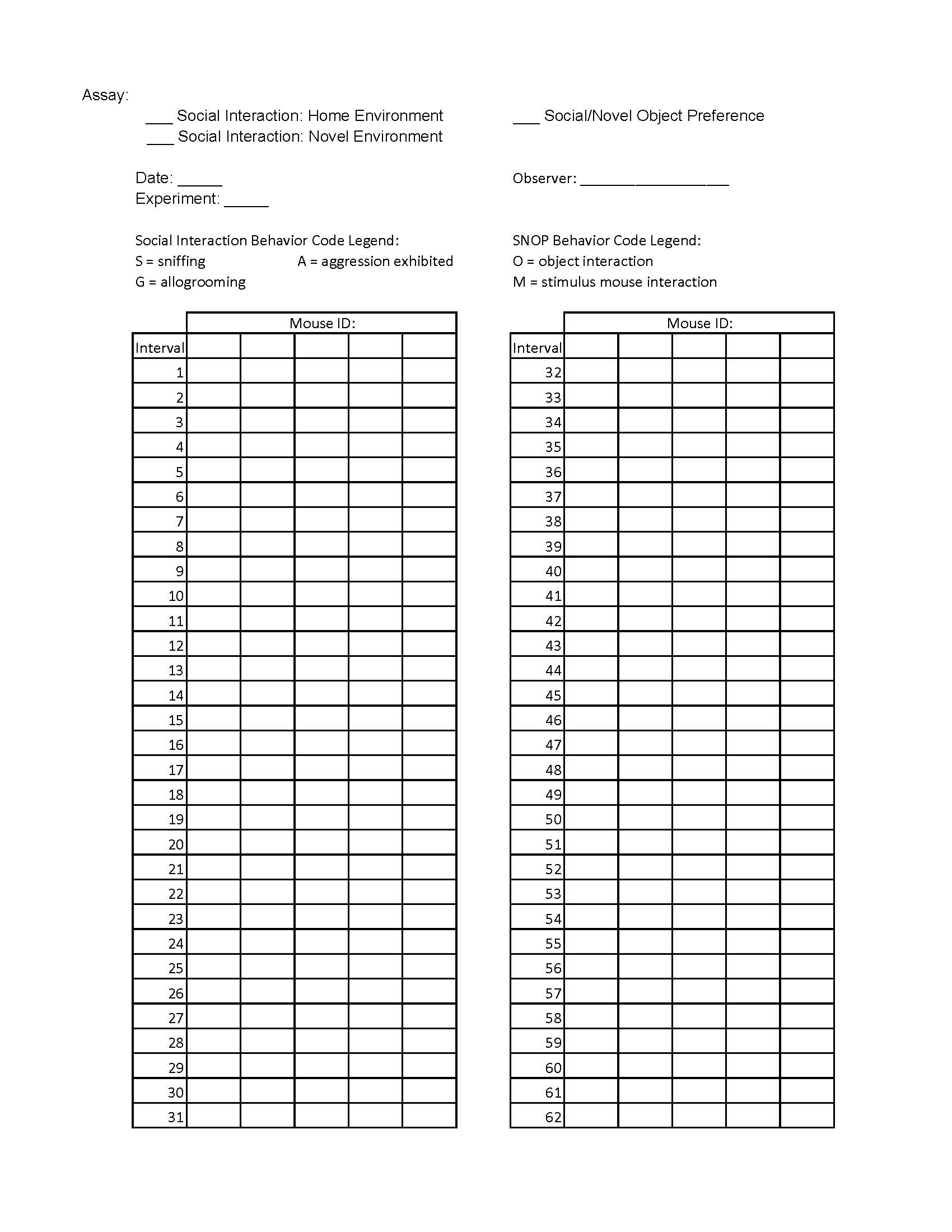
*
