# Supplementary material for: The mouse Social Frailty Index (mSFI): a novel behavioral assessment for impaired social functioning in aging mice
Source: GeroScience. 2024 Jul 11;47(1):85–107. doi: 10.1007/s11357-024-01263-4 (PMC11872866; doi:10.1007/s11357-024-01263-4)
Supplement: Supplementary file 2 — Supplementary file2 (DOCX 144 KB) [file 11357_2024_1263_MOESM2_ESM.docx]

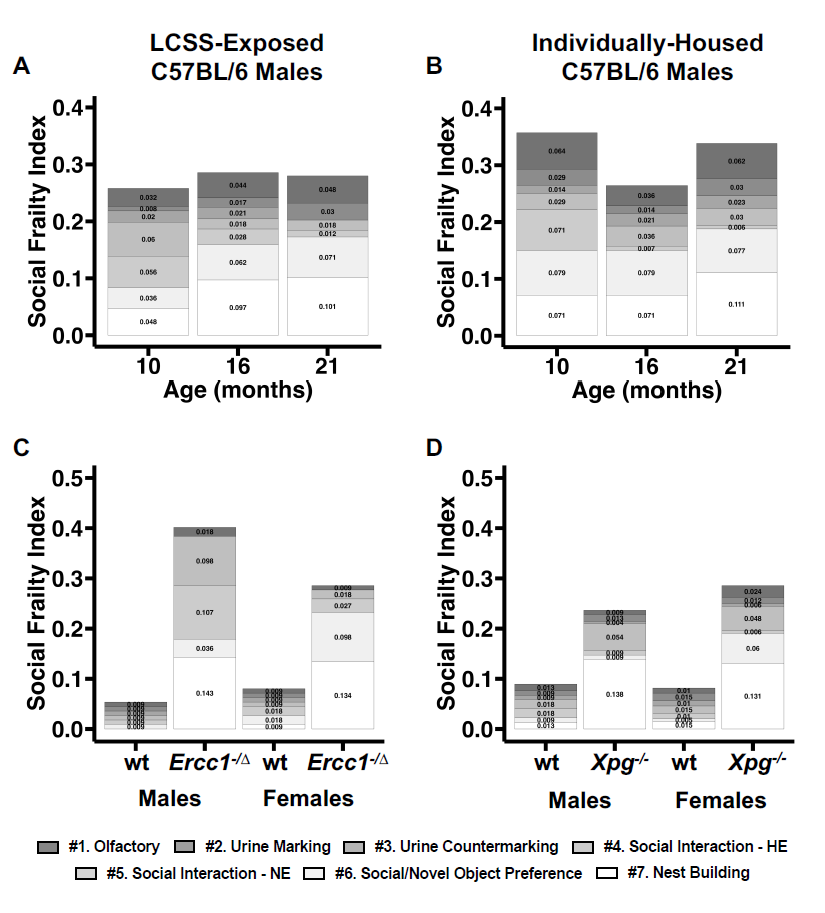


**Supplementary Figure 1**

A) Decomposition of mean mSFI values by contribution from each individual item per age group in LCSS-exposed mice measured at 10, 16, and 21 mo. B) Decomposition of mean mSFI values by contribution from individual items per age group in lifelong individually-housed mice measured at 10, 16, and 21 mo. C) Decomposition of mean mSFI values by contribution from individual items in *Ercc1*^-/Δ^ male and female mice compared to wild-type (wt) littermates at 8 wks. D) Decomposition of mean mSFI values by contribution from individual items in *Xpg^-/-^* male and female mice compared to wild-type (wt) littermates at 8 wks.
