# Supplementary material for: The mouse Social Frailty Index (mSFI): a novel behavioral assessment for impaired social functioning in aging mice
Source: GeroScience. 2024 Jul 11;47(1):85–107. doi: 10.1007/s11357-024-01263-4 (PMC11872866; doi:10.1007/s11357-024-01263-4)
Supplement: Supplementary file 3 — Supplementary file3 (DOCX 55 KB) [file 11357_2024_1263_MOESM3_ESM.docx]

**Supplementary Table 1**

*Performance of Group-Housed C57BL/6 Male Mice in mSFI Individual Items*

| **A** | **Olfactory** | | | **Marking** | | | **Countermarking** | | |
| --- | --- | --- | --- | --- | --- | --- | --- | --- | --- |
| Age | % U.P. | SD | Score | # Mark | SD | Score | # Mark | SD | Score |
| 4 mo. | 63.73 | 20.04 | 0.10 | 47.10 | 39.01 | 0.05 | 9.40 | 10.71 | 0.05 |
| 10 mo. | 33.72^#^ | 10.15 | 0.25 | 51.20 | 31.84 | 0.08 | 15.60 | 17.70 | 0.13 |
| 16 mo. | 45.65 | 18.34 | 0.15 | 37.80 | 51.50 | 0.15 | 61.90 | 59.35 | 0.58^#^ |
| 21 mo. | 58.30 | 20.80 | 0.10 | 79.60 | 56.04 | 0.17 | 208.0^#†/^ | 133.6 | 0.88^#†^ |
| 27 mo. | 57.48 | 30.05 | 0.19 | 132.6^/^ | 91.41 | 0.50^#†^ | 48.13^⦅^ | 39.07 | 0.63^#^ |
| 34 mo. | 26.64^#^ | 24.55 | 0.35 | 137.2 | 114.4 | 0.5^#^ | 165.4^#†^ | 124.8 | 0.80^#†^ |

| **B** | **Social Int. – Home** | | | **Social Int. - Novel** | | | **Social/Novel Preference** | | |
| --- | --- | --- | --- | --- | --- | --- | --- | --- | --- |
| Age | % Int. | SD | Score | % Int. | SD | Score | % Soc. | SD | Score |
| 4 mo. | 35.15 | 12.31 | 0.10 | 25.53 | 9.74 | 0.10 | 39.10 | 17.79 | 0.08 |
| 10 mo. | 22.01 | 11.21 | 0.15 | 13.66^#^ | 8.62 | 0.18 | 12.15 | 24.90 | 0.30 |
| 16 mo. | 41.5^†⦅^^ | 13.44 | 0.10 | 19.58 | 8.85 | 0.13 | 28.13 | 29.16 | 0.23 |
| 21 mo. | 26.80 | 9.66 | 0.10 | 21.92 | 6.02 | 0.05 | 35.59 | 22.47 | 0.17 |
| 27 mo. | 22.86 | 12.30 | 0.19 | 12.05^#^ | 4.15 | 0.22 | 24.81 | 41.30 | 0.44^#^ |
| 34 mo. | 33.00 | 16.05 | 0.15 | 12.12^#^ | 8.83 | 0.25 | 23.67 | 25.81 | 0.25 |

| **C** | **Nest Building** | | |  | **D** | **Overall mSFI** | |
| --- | --- | --- | --- | --- | --- | --- | --- |
| Age | Nest | SD | Score |  | Age | mSFI | SD |
| 4 mo. | 4.90 | 0.32 | 0.03 |  | 4 mo. | 0.07 | 0.06 |
| 10 mo. | 4.10 | 1.29 | 0.23 |  | 10 mo. | 0.19^#^ | 0.08 |
| 16 mo. | 4.60 | 0.70 | 0.10 |  | 16 mo. | 0.20^#^ | 0.06 |
| 21 mo. | 2.87^#/^ | 1.41 | 0.53^#/^ |  | 21 mo. | 0.29^#†^ | 0.06 |
| 27 mo. | 2.88^#/^ | 1.13 | 0.53^#/^ |  | 27 mo. | 0.38^#†/^ | 0.13 |
| 34 mo. | 3.2 | 1.64 | 0.45 |  | 34 mo. | 0.39^#†/^ | 0.09 |

**Supplementary Table 1 Legend.** Results of 4 mo. (N = 10), 10 mo. (N=10), 16 mo. (N = 10), 21 mo. (N = 15), 27 mo. (N = 8), and 34 mo (N =5) group housed C57BL/6 male mice in A) social communication B) social interaction C) social motivation mSFI assays (Score: Index Score for individual item). D) Mean overall mSFI scores for each age group, also presented graphically in Figure 1B. Significance was determined with one-way ANOVA testing and subsequent Tukey HSD post hoc testing (% U.P., % urine preference; % Mark, % area marked; % Int., % interaction; % App., % social approach). #: Indicates significantly different from 4 mo., †: different from 10 mo., /: different from 16 mo., ⦅: different from 21 mo., ^: different from 27 mo. in Tukey HSD.

**Supplementary Table 2**

*Performance of Group-Housed C57BL/6 Female Mice in mSFI Individual Items*

| **A** | **Olfactory** | | | **Marking** | | | **Countermarking** | | |
| --- | --- | --- | --- | --- | --- | --- | --- | --- | --- |
| Age | % U.P. | SD | Score | # Mark | SD | Score | # Mark | SD | Score |
| 4 mo. | 47.97 | 18.28 | 0.07 | 5.43 | 7.39 | 0.05 | 2.71 | 4.25 | 0.07 |
| 10 mo. | 45.43 | 7.29 | 0.00 | 3.70 | 3.16 | 0.00 | 4.10 | 3.96 | 0.08 |
| 16 mo. | 36.51 | 10.65 | 0.08 | 1.30 | 3.20 | 0.00 | 7.80 | 8.10 | 0.23 |
| 21 mo. | 40.35 | 20.50 | 0.10 | 2.70 | 3.37 | 0.00 | 4.60 | 4.53 | 0.13 |
| 27 mo. | 52.27 | 15.01 | 0.08 | 4.58 | 6.39 | 0.06 | 2.58 | 4.72 | 0.06 |
| 34 mo. | 26.33^#^^ | 18.42 | 0.16 | 4.38 | 6.14 | 0.03 | 4.50 | 8.04 | 0.16 |

| **B** | **Social Int. – Home** | | | **Social Int. - Novel** | | | **Social/Novel Preference** | | |
| --- | --- | --- | --- | --- | --- | --- | --- | --- | --- |
| Age | % Int. | SD | Score | % Int. | SD | Score | % Soc. | SD | Score |
| 4 mo. | 34.64 | 15.66 | 0.09 | 13.80 | 6.68 | 0.09 | 14.89 | 38.83 | 0.09 |
| 10 mo. | 33.05 | 13.82 | 0.08 | 19.92 | 6.89 | 0.10 | 0.33 | 29.56 | 0.05 |
| 16 mo. | 33.98 | 12.64 | 0.05 | 16.82 | 6.00 | 0.10 | 13.78 | 39.15 | 0.10 |
| 21 mo. | 25.34 | 12.76 | 0.10 | 13.71 | 8.77 | 0.15 | 10.08 | 31.29 | 0.03 |
| 27 mo. | 27.82 | 15.32 | 0.13 | 15.49 | 6.12 | 0.08 | 34.21 | 31.16 | 0.10 |
| 34 mo. | 25.94 | 18.89 | 0.16 | 11.97 | 6.52 | 0.06 | 24.81 | 35.44 | 0.06 |

| **C** | **Nest Building** | | |  | **D** | **Overall mSFI** | |
| --- | --- | --- | --- | --- | --- | --- | --- |
| Age | Nest | SD | Score |  | Age | mSFI | SD |
| 4 mo. | 4.21 | 0.58 | 0.20 |  | 4 mo. | 0.09 | 0.06 |
| 10 mo. | 4.10 | 0.74 | 0.23 |  | 10 mo. | 0.08 | 0.06 |
| 16 mo. | 4.30 | 0.67 | 0.18 |  | 16 mo. | 0.10 | 0.05 |
| 21 mo. | 2.60^#†/^ | 0.97 | 0.60^#†/^ |  | 21 mo. | 0.16 | 0.08 |
| 27 mo. | 2.58^#†/^ | 1.31 | 0.60^#†/^ |  | 27 mo. | 0.16 | 0.08 |
| 34 mo. | 1.86^#†/^ | 0.99 | 0.78^#†/^ |  | 34 mo. | 0.20^#†/^ | 0.10 |

**Supplementary Table 2 legend.** Results of 4 mo. (N = 14), 10 mo. (N = 10), 16 mo. (N = 10), 21 mo. (N = 10), 27 mo. (N = 12), and 34 mo. (N = 8) group housed C57BL/6 female mice in A) social communication B) social interaction C) social motivation mSFI assays (Score: Index Score for individual item). D) Mean overall mSFI values for each age group, also presented graphically in Figure 2A. Significance was determined with one-way ANOVA testing and subsequent Tukey’s HSD post hoc testing (% U.P., % urine preference; % Mark, % area marked; % Int., % interaction; % App., % social approach). #: Indicates significantly different from 4 mo., †: different from 10 mo., /: different from 16 mo., ^: different from 27 mo. in Tukey HSD.

**Supplementary Table 3**

*mSFI Individual Assay Reference Values Obtained in 8 wk. Ercc1 wt and 8 wk. Xpg wt mice of both sexes*

|  | **Males** | | **Females** | |
| --- | --- | --- | --- | --- |
| **Assay** | *Ercc1* wt | *Xpg* wt | *Ercc1* wt | *Xpg* wt |
| Olfactory | 70.53 $\pm$16.83 | 41.64 $\pm$16.52 | 39.85 $\pm$30.55 | 56.02 $\pm$17.69 |
| Marking | 106.00 $\pm$124.49 | 72.13 $\pm$58.98 | 13.50 $\pm$18.50 | 11.00 $\pm$7.48 |
| Countermarking | 113.75 $\pm$120.61 | 39.63 $\pm$50.47 | 16.00 $\pm$14.24 | 5.57 $\pm$8.10 |
| Soc Int. – Home | 64.71 $\pm$12.06 | 81.21 $\pm$17.70 | 60.64 $\pm$20.62 | 79.62 $\pm$13.50 |
| Soc Int. – Novel | 18.65 $\pm$5.70 | 38.63 $\pm$9.61 | 28.57 $\pm$6.80 | 31.78 $\pm$13.51 |
| SNoP | 29.38 $\pm$21.05 | 36.79 $\pm$33.21 | 18.79 $\pm$10.82 | 32.45 $\pm$13.35 |

**Supplementary Table 3 legend.** Mean $\pm$ SD results; 8 wk. male (N = 4), female (N = 4) *Ercc1* wt (C57BL6/FVB F1); 8 wk. male (N = 8), female (N = 7) *Xpg* wt (C57BL6/FVB F1) mice in all individual assays used as reference values for strain- and sex-matched to assign social frailty via the mSFI. All values presented per each assay are the respective quantitative values obtained from that assay (e.g. % urine preference is obtained from the olfactory test).
